# The Polypill: A New Alternative in the Prevention and Treatment of Cardiovascular Disease

**DOI:** 10.3390/jcm13113179

**Published:** 2024-05-29

**Authors:** Enma V. Páez Espinosa, Eugenia Mato Matute, Delia M. Sosa Guzmán, Fadi T. Khasawneh

**Affiliations:** 1 Department of Clinical Laboratory, School of Medicine, Pontifical Catholic University of Ecuador, Quito 170143, Ecuador; dmsosag@puce.edu.ec; 2 Center for Research on Health in Latin America (CISeAL), Pontifical Catholic University of Ecuador, Quito 170143, Ecuador; 3 Department of Endocrinology and Nutrition, Hospital de la Santa Creu i Sant Pau, 08041 Barcelona, Spain; emato@santpau.cat; 4 Networking Research Centre of Bioengineering, Biomaterials and Nanomedicine (CIBER-BBN), C/Monforte de Lemos 3-5, 28029 Madrid, Spain; 5 Department of Pharmaceutical Sciences, Rangel School of Pharmacy, Texas A&M University, College Station, TX 77843, USA; fkhasawneh@tamu.edu

**Keywords:** polypill, cardiovascular disease, hypertension, LDL cholesterol, randomized clinical trials, efficiency, adherence, cost

## Abstract

Cardiovascular disease (CVD) is the primary cause of death and disability worldwide. Although age-standardized CVD mortality rates decreased globally by 14.5% between 2006 and 2016, the burden of CVD remains disproportionately higher in low- and middle-income countries compared to high-income countries. Even though proven, effective approaches based on multiple-drug intake aimed at the prevention and treatment of CVD are currently available, poor adherence, early discontinuation of treatment, and suboptimal daily execution of the prescribed therapeutic regimes give rise to shortfalls in drug exposure, leading to high variability in the responses to the prescribed medications. Wald and Law, in their landmark paper published in BMJ 2003, hypothesized that the use of a fixed-dose combination of statins, β-blockers, angiotensin receptor blockers, angiotensin-converting enzyme inhibitors, and aspirin (classic Polypill composition) may increase adherence and decrease CVD by up to 80% when prescribed as primary prevention or in substitution of traditional protocols. Since then, many clinical trials have tested this hypothesis, with comparable results. This review aims to describe the available clinical trials performed to assess the impact of fixed-dose combinations on adherence, cost-effectiveness, and the risk factors critical to the onset of CVD.

## 1. Introduction

Cardiovascular disease (CVD) is a multifactorial group of disorders of the peripheral vessels, leading to cardiac manifestations such as ischemic heart disease (IHD), myocardial infarction, or stroke. The latest reports on heart disease and stroke statistics indicate that CVD was the most common underlying cause of death in the world in 2020, accounting for an estimated 19 million deaths [1], with 80% of CVD mortality originating from lower-income countries [2].

CVD represents an important economic burden. From 2017 to 2018, it was responsible for 12% of total US health expenditures—more than any major diagnosed disease group. Survey data from 1999 to 2012 found increased use of lipid-lowering, antihypertensive, and antidiabetic medications over time in developed countries, which resulted in a combined 51% reduction in individual expenses related to CVD. The latter was due to the decrease in hospitalizations and/or other major heart procedures that are a consequence of cardiovascular complications [3]. Despite this price reduction trend, the newest reports indicate that cost-effective medications such as aspirin, statins, and blood pressure (BP)-lowering agents remain unaffordable for much of the world [2], indicating that novel approaches are necessary to improve the health outcomes of individuals at risk at a global level.

There are multiple strategies for the prevention and treatment of CVD based on lifestyle and pharmacologic interventions. Unfortunately, the culture and level of instruction and motivation can compromise the efficacy of schemes based on dietary or life-change regimens [4]. Although the pharmacological approach based on aspirin, antihypertensive drugs, and statins reduces the incidence of CVD in the population and lowers acute cardiovascular events in high-risk patients with already established CVD [5,6], the efficacy of this approach is hindered by several issues, such as inadequate prescription, poor adherence to treatment, limited availability of medications, and the unaffordable cost of the treatment.

The first Polypill was developed more than two decades ago by Nicholas Wald and Malcolm Law of the Wolfson Institute. It was created to provide a therapeutic option that could enhance patient adherence and improve treatment outcomes. The pill was a combination of medications already available in the market to address CVD, ischemic heart disease, and stroke. It came in the form of a single tablet that was taken once a day, with doses designed to maximize the effectiveness of each component, minimize adverse effects, and reduce the cost of treatment [7]. The formulation of the Polypill was the result of a series of studies performed by the group with the aim of including categories of drugs or vitamins used in medical practice for more than 10 years with substantial evidence on safety and efficacy to modify LDL cholesterol (LDL-C), BP, homocysteine, and platelet function [7].

The researchers used a simple Markov model stratified by sex and age to estimate the effect of the Polypill on the combined outcome of changing the four risk factors of CVD over time. The results showed an overall 88% reduction in ischemic heart events and an 80% reduction in stroke, directly benefiting 30% of the population at risk of developing CVD, as well as those who already had established diagnoses. The study also estimated that using the Polypill could increase an individual’s life expectancy by 11–12 years without experiencing a heart attack or stroke [7].

Since then, several Polypill formulations have been manufactured and assessed in clinical trials for the prevention and treatment of CVD. The purpose of this review is to discuss the most recent research on the composition, development, safety, and impact of the Polypill in clinical practice, as well as the potential implications of this approach in the prevention and treatment of CVD, including the medical outcome, adherence, cost of the treatment, and commitment of the medical community to include the Polypill in their prescriptions.

## 2. Materials and Methods

The present work followed the PRISMA Preferred Reporting Items for Systematic Reviews and Analyses [8].

### 2.1. Search Terms

We searched the PubMed (National Institute of Health, Bethesda, MD, USA), Scopus (Elsevier BV, Amsterdam, The Netherlands), and Cochrane databases for relevant studies published in English from 2003 to 2023. The search terms included were “polypill” AND “cardiovascular disease AND/OR Randomized Clinical Trial AND/OR Hypertension AND/OR Systematic Reviews OR Meta-analysis”. 

### 2.2. Search Strategy and Inclusion Criteria

We conducted a review of multiple studies and trials that evaluated the use of pharmacological treatments for the prevention of cardiovascular disease. This included observational, cross-sectional studies; registries; randomized controlled clinical trials; randomized factorial trials; systematic reviews; and meta-analyses. We did not set any specific criteria for the sex or geographic origin of the study participants, but we did consider factors such as race and economic status. We excluded case reports and editorials. After analyzing the full text of relevant articles, we checked their references for additional suitable studies that met our inclusion criteria and contributed to a better understanding of the Polypill principle in current medical practice. 

### 2.3. Evaluation of the Studies

The JBI critical appraisal checklist for narrative, expert opinion, and textual evidence was used to evaluate the search results [9]. The tool provides a score on eight items, allowing for quick visual assessment (see Supplementary Material S1).

### 2.4. Results 

**Figure 1:**
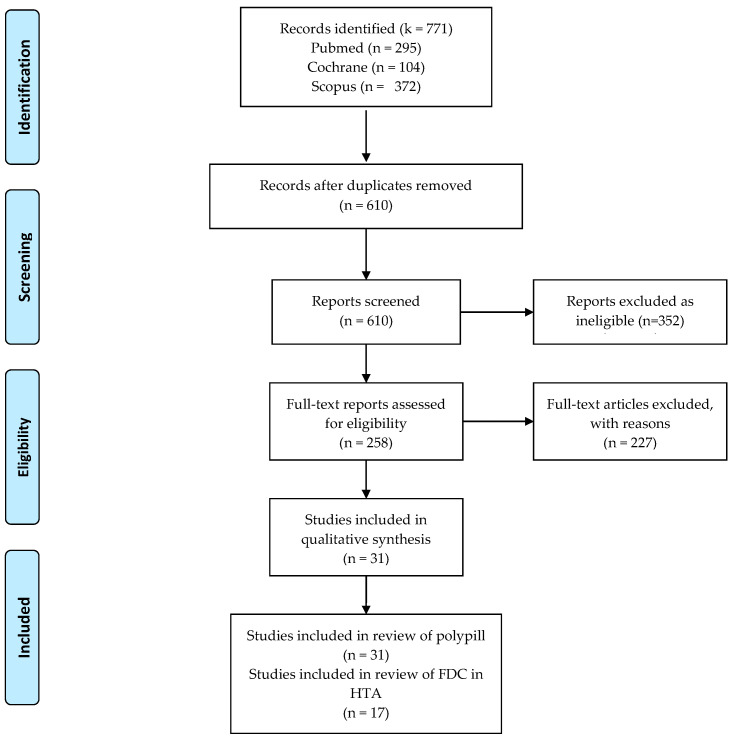
PRISMA flow diagram. From: Moher D, Liberati A, Tetzlaff J, Altman DG, The PRISMA Group (2009). Preferred Reporting Items for Systematic Reviews and Meta-Analyses: The PRISMA Statement. PLoS Med 6(6): e1000097. doi:10.1371/journal/p.med1000097 [8]. n stands for the number of studies.

A search of the databases yielded a total of 771 records. After removing duplicates, we were left with 610 results. Out of these, we assessed 258 records for eligibility. Finally, we selected 31 studies that reported on the effectiveness, adherence, safety, accessibility, cost, composition, and acceptance of the Polypill for primary and secondary prevention of CVD-related events in high-risk individuals. Furthermore, we incorporated 17 studies that discussed the use of fixed-dose combination (FDC) pills to treat hypertension. These investigations evaluated parameters that were identical to those previously reported. We incorporated FDC tablets into our analysis due to their existing availability in the market and the regulatory permission they have obtained in the countries where they are sold (Figure 1).

## 3. The Polypill: A Brief History

The concept of the Polypill was developed as an alternative to the traditional multi-drug treatment for CVD with the aim of improving medical outcomes by increasing individuals’ adherence to treatment and reducing the overall cost of pharmacologic treatment [10,11,12,13].

To determine which components to include in the original Polypill, Wald and Law calculated the relative risk reduction offered by each component. The first formulation of the Polypill included one of three statins (10 mg atorvastatin, 40 mg lovastatin, or 40 mg simvastatin) and three BP-lowering drugs at half the standard dose (a thiazide, a β-blocker, an angiotensin-converting enzyme inhibitor (ACE), low-dose aspirin (75 mg), and folic acid (0.8 mg)) [7]. BP-lowering drug categories and doses were determined through a meta-analysis of short-term randomized trials [13,14]. Statins were selected for their ability to lower LDL-C by up to 37% [13]. Aspirin was included after weighing its benefits against the risk of bleeding in high-risk patients [7]. The presence of folic acid in the original design of the pill was supported by studies showing that lowering serum homocysteine levels with folic acid provided additional reduction in the risk of CV events due to coronary artery disease (CAD) [15,16,17].

After the success of the first Polypill, several fixed-dose combinations (FDCs) were produced and assessed to test their effect on cholesterol and BP reduction in well-controlled clinical studies. Newer Polypills introduce variations in the doses and combinations of antihypertensives, statins, and platelet inhibitors, considering the target populations, comorbidities, resistance, or hypersensitivity to any of the drugs present in the formulation. This has generated a variety of pills whose components act over several risk factors of CVD known as multipurpose Polypills. Other investigators applied the FDC concept to produce a pill made of very low doses of different compositions aimed to prevent just one cardiovascular (CV) risk factor, called a single-purpose Polypill [18].

The underlying principle applied by Wald and collaborators for the design of the first Polypill is considered a good model to gain greater adherence to CVD treatment and optimize the cost/benefit ratio at both the individual and community levels. The Polypill is still a subject of investigation to find better combinations that respond to the necessities of each particular risk of CVD of the individual.

## 4. The Rationale behind the Use of the Polypill in the Management of Hypertension and Hyperlipidemia for the Prevention and Treatment of Cardiovascular Diseases

### 4.1. Blood Pressure and Cardiovascular Risk

Hypertension is the most common cardiovascular disorder in the world. According to the World Health Organization (WHO), it affects 1.28 billion adults aged 30–79 years globally, with two-thirds living in low- and middle-income countries [19]. In 2019, the average prevalence of hypertension in adults aged 30–79 years was reported to be 34% in men and 32% in women [20].

Hypertension is defined as repeated office systolic blood pressure (SBP) values of over 140 mmHg and/or diastolic blood pressure (DBP) of over 90 mmHg. However, research has shown that the risk of cardiovascular or renal events increases continuously from an office SBP of over 115 mmHg and a DBP of over 75 mmHg. These BP values correspond to the levels of BP at which the benefits of intervention exceed those of inaction, as shown by outcome-based randomized controlled trials (RCTs) [21,22]. Prospective studies on cardiovascular morbidity and mortality based on several indicators of CV risk found that pre-hypertension levels of 120–139/80–89 mmHg are already associated with increased risk of CVD, with reports that pre-hypertension (pre-HT) is linked to 45% higher risk of CVD events than normal BP after adjusting for traditional CVD risk factors [23]. The progression from pre-HT to hypertension entails ongoing functional and structural vascular alterations, leading to tissue perfusion disorders and susceptibility to ischemia, the hallmark of CVD [24,25]. If hypertension is left untreated, it produces CV changes in the heart, kidneys, and arteries, leading to left ventricular hypertrophy, microalbuminuria, and vascular damage, which further increase the CV risk and the occurrence of fatal CV events [26,27].

Achieving the goal of hypertension treatment requires a two-pronged approach of modifying lifestyle and prescribing antihypertensive drugs to reduce BP to <130/80 mm Hg [28]. While maintaining a healthy lifestyle is important, it is difficult to comply. For that reason, most patients need medications to control their BP. First-line drugs including thiazide diuretics, CCBs, and ACEIs/ARBs must be prescribed in adequate number and dose. For stage 2 hypertension, at least two first-line drugs are recommended with an average BP of 20/10 mm Hg above the BP target. However, low adherence, high prevalence of uncontrolled BP, increased risk for CVD events, and excess healthcare costs continue to pose a challenge [22,28,29].

To examine whether early pharmacological treatment in subjects with “high–normal” BP might prevent or delay the development of clinical hypertension, interventional studies such as the TROPHY (Trial of Preventing Hypertension) [30], PHARAO (Prevention of Hypertension with the ACE-inhibitor Ramipril in Patients with High Normal Blood Pressure) [31], and CALIBER (CArdiovascular research using LInked Bespoke studies and Electronic health Records) studies, as well as systematic reviews and meta-analyses of RCTs [32,33], provide suitable evidence that antihypertensive treatments reduce cardiovascular events and all-cause mortality.

A promising new way to manage high BP and reduce side effects was proposed in the QUARTET study, which was conducted in Australia and involved adult patients with hypertension. The principle that is currently recommend by the WHO [34] consists of combining low doses of two to four antihypertensive drugs. Participants were randomly assigned to receive either a single pill containing 150 mg irbesartan or a combination pill containing ultra-low doses of four different BP-lowering medications (37.5 mg irbesartan, 1.25 mg amlodipine, 0.625 mg indapamide, and 2.5 mg bisoprolol), demonstrating the simplicity, tolerability, and effectiveness of a quadpill-based strategy compared with the common strategy of initial standard-dose monotherapy [35,36].

The trial followed the evidence provided by the systematic review and meta-analysis of 36 RCTs (n = 4721) of one drug at quarter dose and 6 trials (n = 312) of two drugs at quarter dose against placebo conducted in 2017 by Bennett et al. [37]. In this study, the authors reported larger BP reductions of quarter-dose combination therapy for hypertension in comparison to standard-dose monotherapy. Although the authors provided little data on tolerability the meta-analysis did not compare results with guideline-recommended therapy of up-titration if BP is not con-trolled after initial monotherapy. However, the study was well-designed, used standard dosages, and stressed the validity of the results [37].

The QUARTET trial recruited 591 participants with a mean age of 59 years (SD, 12); 356 (60%) were male, and 235 (40%) were female; 483 (82%) were White, 70 (12%) were Asian, and 38 (6%) were reported as another race or ethnicity, providing valuable information regarding sex and ethnicity. The components of the quadpill were very low doses of medications commonly prescribed in Australia. The pill was intended to be taken in single daily dosing and was tested against one monotherapy control. Each component was at a quarter of the standard dose, defined as the usual maintenance dose recorded by the British National Formulary, Martindale, and the Monthly Index of Medical Specialties. BP changes were assessed at 12 weeks and 12 months. The adequate design and execution of this study demonstrate the efficacy of this approach in any ethnic group and gender, as well as the suitability of the formulation of one pill with four different components [35].

Nevertheless, within the field of clinical practice, the efficacy rate of these interventions has remained low. Evidence indicates that trials are carried out in a meticulously regulated setting to optimize adherence to the prescribed treatment regimen and minimize therapeutic inertia, which refers to the failure to adjust the dosage or change treatment in the case of ineffectiveness [38]. However, individuals who are not part of a trial do not adhere to these stringent regulations. Indeed, numerous global surveys have demonstrated that a considerable percentage of individuals engaged in primary and secondary cardiovascular disease (CVD) prevention struggle to attain the desired levels of blood pressure (BP), lipids, and glucose as advised by existing guidelines. This lack of adherence and the presence of high therapeutic inertia contributes to the difficulty in achieving optimal disease control for a significant portion of patients [39,40].

To enhance adherence and overcome the poor response to antihypertensive therapy, RCTs comparing two different drug combinations have been performed. In most trials, treatment was initiated using a single medication, and another drug was added later, usually in a nonrandomized way. Trials such as ALLHAT [41] have evaluated whether a calcium channel blocker or an angiotensin-converting enzyme inhibitor lowers the incidence of coronary heart disease (CHD) or other cardiovascular (CV) events vs. treatment with a diuretic, precluding the use of optimal combinations in a single pill but opening the way to the study of such combinations in a single pill. Since then, a variety of drug combinations have been used in at least one active arm of placebo-controlled trials and have been associated with significant benefit with respect to major CV events [22].

The 2023 ESH Guidelines for the management of arterial hypertension summarized the most significant drug combinations tested in various trials in different risk populations, either using a stepped approach or a randomized combination against placebo or monotherapy [22]. The association of diuretics with antihypertensive agents was tested in the PROGRESS, ADVANCE, and HYVET trials (ACEi); the SCOPE and HOPE-3 trials (ARBs); and the FEVER trial (CCB), while Coope and Warrender, SHEP, and STOP-Hypertension 1 and 2 trials included BB in their trials. Likewise, the combination of two types of antihypertensives was analyzed in the OSCAR (ARB/CCB), Syst-Eur, Syst-China (ACEi/CCB), and INTARGET or ALTITUDE (two RAS blockers/ACEi + ARB or RAS blocker + renin inhibitor) trials [22]. All of them showed a significant decrease in SBP regarding the controls and were associated with positive outcomes on major CV events [22].

Those results encouraged researchers to formulate and test single pills containing two combinations of antihypertensives and diuretics in varying proportions and compare their effects in randomized trials. These combination therapies showed similar benefits, except for two trials, where patients received either an ARB/diuretic combination (the Losartan Intervention For Endpoint reduction in hypertension study (LIFE) [39] or a CCB/ACEi combination (the Anglo-Scandinavian Cardiac Outcomes Trial-Blood Pressure Lowering Arm (ASCOT-BPLA) [42], which were superior to a BB (atenolol)/diuretic combination in reducing cardiovascular outcomes. Only in the ACCOMPLISH study was the ACEi/CCB combination superior to the same ACEi in combination with a thiazide diuretic at preventing major cardiovascular outcomes and chronic kidney disease (CKD) progression, despite only a small difference in BP between the two arms.

The outcomes of the RCTs indicate that initial two-drug combination therapy acts faster than monotherapy, reduces the heterogeneity of the BP response to initial treatment, diminishes treatment inertia, enhances adherence, and is safe and well tolerated [27,28,29]. Further evidence showed that two-drug combination therapy controls BP in half to two-thirds of patients, while a three-drug combination can control BP in up to 90% of patients [30,31]. Currently, it is recommended that hypertension treatment be based on combinations of an ACEi or an ARB with CCB or a thiazide/thiazide-like diuretic, avoiding the combination of ACEis and ARBs [22]. Approved combination pills and their therapeutic alternatives are listed in the latest WHO Model List of Essential Medicines [34]. Table 1 shows some of the more representative studies on fixed-dose combination (FDC) pills for hypertension treatment and their principal outcomes.

### 4.2. Hyperlipidemia and Cardiovascular Risk

CVDs are the leading cause of morbidity, premature deaths, and mortality around the world. The major medical risk factors associated with CVD are high BP, high total cholesterol levels, high BMI, and high fasting plasma glucose. High blood pressure (BP) and high total cholesterol levels have the highest average impact on the burden of cardiovascular disease (CVD), accounting for 56% and 30% of deaths, respectively, with high BMI and hyperglycemia accounting for less than 20% [56,57].

Atherosclerosis is, by far, the most important source of CVD. It has a multifactorial origin, including non-modifiable risk factors and other factors that can potentially be modified through lifestyle modifications and pharmacological approaches, like hypertension, dyslipidemia, diabetes, or a prothrombotic state [58]. The presence of multiple cardiovascular risk factors is common in patients with established atherosclerotic cardiovascular disease (ASCVD). Therefore, treating these patients usually involves targeting these risk factors with appropriate therapies. Guidelines recommend the use of low-dose acetylsalicylic acid (ASA), angiotensin-converting enzyme inhibitors (ACEis), and statins to reduce the occurrence of major adverse cardiovascular events (MACEs) in patients with established ASCVD [22,59].

Dyslipidemia is the primary cause of atherosclerosis, with LDL-C being the major cholesterol source that contributes to the development of atherosclerotic plaque. Conversely, the concentration of HDL-C inversely correlates with the risk of CVD. Although some lifestyle modifications can help regulate the excess of LDL-C, they are often insufficient, and medical treatments become necessary to maintain a balance between LDL-C and HDL-C. Statins are HMG-CoA reductase inhibitors that induce significant reductions in total cholesterol and LDL-C levels. They also decrease the incidence of myocardial infarction, stroke, and CVD in individuals with and without ASCVD, as well as all-cause mortality in higher-risk patients [60,61,62]. Furthermore, statins have anti-inflammatory and direct effects on the plaque, leading to coronary plaque stabilization and even modest regression of atheroma, a property that can be useful to prevent cardiovascular events in patients with minimal risk of developing the disease [63,64]. Unfortunately, doctors tend to under-prescribe or under-dose these drugs, mostly out of fear of side effects [59].

To address this issue, several studies have been conducted. Although previous studies reported adverse effects in patients treated with statins for a long time [65], newer observational studies and meta-analyses based on comparisons of the efficacy, tolerability, and harm of long-term use of statins showed strong evidence that statins, as a class, are generally safe and that the absolute excess risk of the harmful unintended effects is very small compared to the beneficial effects with respect to major cardiovascular events [66,67,68,69].

The Cholesterol Treatment Trialists (CTT) Collaboration, was one of the biggest meta-analyses of individual data from 170,000 participants in 21 trials involving standard statin regimens versus controls and five trials of more intensive versus less intensive regimens [70]. The study concluded that the reduction in LDL-C with a statin decreases the risk of both major vascular events and all-cause mortality (RR 0.79 per 1.0 mmol/L reduction), irrespective of age, sex, baseline LDL-C, or previous vascular disease. The proportional reduction in major vascular events was at least as big in the lowest- and the higher-risk categories (RR 0.62 per 1.0 mmol/L reduction from lowest to highest risk). These results are similar to what was reported in other trials [71,72], indicating that lowering LDL-C by 1 mmol/L with a standard statin regimen reduces the incidence of major vascular events (MACE) by around a fifth, with a comparable drop in the occurrence of heart disease and stroke resulting from a reduction in LDL-C levels among patients with a low risk of cardiovascular disease (CVD), as observed when the initial risk was 20% [62].

Recent research substantiates and strengthens those conclusions. Recent advancements in understanding the molecular mechanisms behind CVD prompted the development of protocols for the targeted administration of various types of statins. These protocols aim to achieve improved outcomes while minimizing adverse effects, considering factors such as the patient’s cardiovascular risk category, LDL-C target, age, and comorbidities [59]. Moreover, in accordance with the most recent guidelines from the European Society of Cardiology (ESC), it may be deemed appropriate to initiate statin therapy for primary prevention at the age of 70 in individuals with a significantly elevated risk of cardiovascular complications. However, it is important to take into account various factors, including risk modifiers, frailty, projected long-term benefits, comorbidities, and patient preferences [73].

In recent decades, numerous lipid-lowering treatments used both alone and in combination with statin therapy and lipid-targeting gene therapy have been developed. These advancements have fundamentally transformed the approach to treating dyslipidemia using drugs like bile acid sequestrants, fibrates, nicotinic acid, ezetimibe, bempedoic acid, volanesoren, evinacumab, and PCSK 9 inhibitors evolocumab and alirocumab. Emerging gene-based therapy includes small interfering RNAs, antisense oligonucleotides, adeno-associated virus vectors, CRISPR/Cas9-based therapeutics, and non-coding RNA therapy [74]. However, statins continue to be the gold-standard therapy for the primary and secondary prevention of CVD and are pleiotropic. Current guidelines recommend a personalized statin treatment rather than under-prescription, and the complexity and comorbidities of the patient should be considered to provide treatment that best balances the risks and benefits. The current guidelines advocate for customized statin therapy instead of prescribing too many statins, aiming to provide the treatment that best balances the risks and benefits [59,73].

## 5. Current Guidelines for the Management of Hypertension and Dyslipidemia

Epidemiological studies have shown that risk factors tend to occur together in individuals. For example, 80% of hypertension cases also present other CVD risk factors, such as dyslipidemia and diabetes. The presence of multiple risk factors in an individual requires a multifactorial approach, which may involve complex medication regimens and the prescription of a high number of different drug classes [57]. However, despite the effectiveness of pharmacotherapy for secondary prevention, patient adherence to secondary prevention medications is around 50% [75,76], a lack of adherence that has been associated with poorer outcomes [77].

Since the first formulation of the Polypill in 2003 [7], the Polypill strategy has been shown to improve medication adherence and treatment outcomes [57,76,78]. This approach is now recognized by several clinical guidelines, including the current European and US hypertension guidelines, the European guidelines for the management of myocardial infarction, and the European Society of Cardiology (ESC) guidelines for the prevention of CVD [57,58,78,79]. However, the role of aspirin in primary CVD prevention remains controversial due to potential benefits limited by an increased bleeding risk [80,81].

The controversy arises from the findings of several systematic reviews and meta-analyses of RCTs in separate groups of patients (age, comorbidities, and CV risk) that reported similar or no reduction in CV outcomes in low-risk individuals, with a risk of major bleeding consistent across them [70,82]. The largest study to address this issue was performed by Zheng and Alistair (2019), who analyzed a total of 13 trials randomizing 164,225 participants with 1,050,511 participant years of follow-up. The median age of trial participants was 62 years (range, 53–74). Of them, 77,501 (47%) were men, 30,361 (19%) had diabetes, and the median baseline risk of the primary cardiovascular outcome was 10.2% (range, 2.6–30.9%). The results showed that the use of aspirin in patients without CVD was associated with a 38% reduction in risk of cardiovascular events and a 0.47% higher risk of major bleeding [81].

Based on the evidence provided by the clinical trials, the task force for the management of arterial hypertension of the European Society of Cardiology (ESC) and the European Society of Hypertension (ESH), in the 2018 ESC/ESH guidelines for the management of arterial hypertension, recommended low-dose aspirin only for secondary and not primary prevention [5,83]. The 2019 ESC Guidelines on diabetes, pre-diabetes, and cardiovascular diseases developed in collaboration with the EASD also recommends low-dose-aspirin as a routine preventive for primary prevention of atherosclerotic CVD in adults at high risk aged 40–70 years and without risk of bleeding [84].

These recommendations are supported by the findings of a Cochrane systematic review on antiplatelet agents and anticoagulants for hypertension published in 2022 by Shantsila and collaborators. The study included 61,015 patients distributed in six trials. Of them, four studies compared low-dose aspirin versus placebo and found no evidence of a difference in all-cause or CV mortality. The results indicated that aspirin treatment reduced the risk of all nonfatal CV events, albeit increasing the risk of major bleedings, but did not provide conclusive evidence that antiplatelet therapy has a protective effect in hypertensive patients in the setting of primary prevention [85].

On the other hand, while efficacy clinical trials have consistently shown the ability to produce significant drops in blood pressure, post-trial monitoring indicated that these differences gradually decreased until there was no longer any difference in SBP approximately 5 to 6 years after the trial’s termination, suggesting that the current model of care is not producing satisfactory rates of hypertension control anywhere in the world. The more intensively treated arms in the ACCORD (Action to Control Cardiovascular Risk in Diabetes) and SPRINT (Systolic Blood Pressure Intervention Trial) trials achieved sustained SBP reductions of 14.2 and 16.2 mm Hg, respectively, compared with the standard care arms after treatment titration [86,87,88].

Better blood pressure control for adults with uncomplicated hypertension appears to have been achieved in implementation trials and routine practice settings when using the 2023 ESH Management of Arterial Hypertension Guidelines, which recommend a model that includes elements of accurate and precise blood pressure measurement; health promotion; easy access to a knowledgeable, community-based, patient-centered healthcare team; and the use of simple, evidence-based protocols for lifestyle counseling and antihypertensive drug treatment. Reliability in accessing effective and affordable antihypertensive medications is also recommended, with a preference for the use of single-pill combinations for combination drug therapies [22,88].

Currently, there are various versions of the cardiovascular Polypill available in the market consisting of a statin, an antiplatelet drug (usually aspirin), and an antihypertensive agent (ACEi/ARB/Thiazide/β-blocker/CCB) in minimal concentrations to achieve clinical efficiency [89]. Of them, the CNIC (Centro Nacional de Investigaciones Cardiovasculares, Ministerio de Ciencia e Innovación, España) Polypill is the only Polypill containing low-dose aspirin approved by the European Medicines Agency (EMA) and marketed in Europe for primary and secondary prevention of CVD [57,90].

The NEPTUNO study was a retrospective analysis of an anonymized medical history dataset covering patients contained in a previously validated administrative database (the BIG-PAC^®^ database) during the years 2015 to 2018 [91]. The study assessed the effectiveness of the CNIC-Polypill compared to other medications in reducing the incidence of MACE and controlling risk factors in patients with established atherosclerotic cardiovascular disease (ASCVD) [58]. The study included adults diagnosed with coronary heart disease (CHD), cerebrovascular disease, or peripheral artery disease (PAD) who began secondary intervention treatment between January 2015 and December 2018. The patients were divided into four cohorts, namely CNIC-Polypill, monocomponents, equipotent medication, and other therapies. The primary endpoint was the incidence of recurrent MACE within two years of follow-up in all cohorts. Secondary endpoints included time to first recurrent cardiovascular event or death, BP and LDL-C control, and persistence to therapy [58].

After a period of 2 years, it was observed that the CNIC-Polypill cohort showed a lower incidence of recurrent MACE compared to the three control cohorts (19.8% vs. 23.3%, 25.5%, and 26.8%; *p* < 0.001). The most commonly occurring cardiovascular events were ischemic heart disease (44%), followed by PAD (30%) and cerebrovascular disease (26%). After the follow-up period, the decreases in SBP and DBP were significantly higher in the CNIC-Polypill cohort compared to the respective control cohorts (*p* < 0.001 for systolic and diastolic BP). It was also observed that the proportion of patients with controlled BP at the end of the study was significantly higher in the CNIC-Polypill cohort compared to each of the other cohorts (*p* < 0.01 in the CNIC-Polypill cohort compared to the control groups) [58].

Likewise, the investigators observed that although all cohorts experienced a significant reduction in their lipid profiles, including total cholesterol, LDL-C, and triglycerides when compared to baseline, the CNIC-Polypill cohort showed a remarkable reduction in total cholesterol, LDL-C, and triglycerides (*p* < 0.001) when compared to each of the control cohorts, with a significant increase in adherence in the CNIC-Polypill group compared to all other cohorts. It is noteworthy that there were no reports of differences in the risk of bleeding between the groups [58].

The research revealed that the CNIC-Polypill could assist in reaching cardiovascular risk factor targets akin to those recommended by the monotherapy in the 2021 ESC prevention guidelines. Despite potential limitations, a retrospective design is deemed optimal for assessing the efficacy of a treatment approach in real-world clinical settings, given the absence of targeted interventions during the trial. Data extracted from patients’ electronic medical records might have led to the underdiagnosis of certain variables; however, the enrollment of a substantial patient cohort helped mitigate this potential bias [90]. Moreover, the BIG-PAC^®^ database has been validated in previous studies, which enhances the quality of the sample [58]. Although the findings of this study can only be generalized to patients with similar clinical profiles and healthcare management, analysis by average treatment effect (ATE) was performed, which is considered acceptable to demonstrate comparability between cohorts. Furthermore, all patients, except those who died, were followed for 2 years in all study cohorts, and no heterogeneity was observed between them. However, because patients were not randomly assigned to different treatment arms, only indirect causality can be suggested.

To overcome this limitation, a prospective study was conducted to evaluate the effectiveness of a Polypill strategy named CNIC-Polypill. The study aimed to determine whether this strategy, which combines aspirin (100 mg), ramipril (2.5, 5, or 10 mg), and atorvastatin (40 mg), is effective in reducing major cardiovascular events such as cardiovascular death, nonfatal myocardial infarction, nonfatal ischemic stroke, and urgent revascularization. The study was conducted in older patients who had recently experienced a myocardial infarction. The SECURE trial was a phase 3 clinical trial conducted for this purpose [79].

About 113 locations in Spain, Italy, France, Germany, Poland, the Czech Republic, and Hungary hosted the trial. According to current European Society of Cardiology guidelines [92], the participants were randomly assigned to either standard therapy or a Polypill strategy. The patients were either older than 75 years old or at least 65 years old and had at least one of the following risk factors: diabetes mellitus, mild or moderate kidney dysfunction (GFR 30–60 mL/min/1.73 m^2^), previous myocardial infarction, previous coronary revascularization, or previous stroke. Cardiovascular death, nonfatal myocardial infarction, nonfatal ischemic stroke, and urgent revascularization were among the main outcomes. A composite of cardiovascular death, nonfatal type 1 myocardial infarction, and nonfatal ischemic stroke served as the primary and secondary end outcomes [79].

An interesting characteristic of this study was the possibility of performing a medical titration of atorvastatin or ramipril according to the clinical evolution and/or the blood tests results based on the observations recorded throughout the trial. There were follow-up visits at months 6, 12, and 24 and further phone follow-ups at 18, 36, and 48 months. Every appointment included taking a blood sample after fasting and recording blood pressure. The eight-item Morisky Medication Adherence Scale was used to examine adherence at 6-month and 24-month intervals [93]. The Treatment Satisfaction Questionnaire for Medication was used to measure treatment satisfaction at baseline and 24 months later [94]. The SECURE trial was designed to acquire accurate results on the efficacy and safety of the pill, as well as the compliance of the participants with the therapeutic regime, to validate the clinical benefit and the safety of the Polypill.

The results showed a lower risk of major adverse CV events in the group receiving a Polypill than the group receiving the usual care strategy, regardless of country; age; sex; and the presence or absence of comorbidities such as diabetes, CKD, and previous revascularization, and were similar for both primary and secondary outcomes. The results are valid for the general population because the average age at the time of a first myocardial infarction is now 65.6 years for men and 72 years for women, which correspond to the ages of the participants [1].

Regarding safety, 404 out of 1237 patients (32.7%) in the Polypill group experienced adverse events, which is similar to the usual-care group, where 388 out of 1229 patients (31.6%) experienced adverse events. The Polypill group did not show an increase in bleeding incidents, and the incidence of nonfatal major adverse events was 19.2% in the Polypill group and 18.2% in the usual-treatment group, which is consistent with the NEPTUNO research [58]. Additionally, studies that compared antiplatelet medication to a placebo in similar populations demonstrated a relative risk reduction of 20% or more [95]. Considering that the CNIC-Polypill has higher aspirin adherence, this effect could be enhanced.

The quality of the evidence from large RCTs over the years showing that the use of these combinations is associated with reduced risks of CV events in primary and secondary prevention, as well as with improved adherence and quality of life with accessible prices, prompted the WHO Expert Committee to include FDC for prevention of ASCVD in primary and secondary settings in the WHO Essential Medicines List (2023) [34]. A square box designates the component combinations, denoting that additional medications from the corresponding pharmacological categories are available as therapeutic options. This aligns with the existing square-box listings for hydrochlorothiazide, antihypertensives, and statins [34]. According to the World Health Organization (2023), the suggested combinations of Polypills are Aspirin + Atorvastatin + Ramipril, Aspirin + Simvastatin + Ramipril + Atenolol + Hydrochlorothiazide, and Atorvastatin + Perindopril + Amlodipine. [34].

Currently, there is enough evidence showing that the CNIC-Polypill meets all the requirements of efficiency, safety, and adherence in the setting of primary and secondary prevention of CVD. The CNIC-Polypill is now considered safe and clinically effective in treating targeted diseases, taking the exposed patients’ attributes into account.

## 6. The Polypill in the Primary Prevention of Cardiovascular Disease: The Age Factor

When Wald and Law described, for the first time, the Polypill as a new strategy to prevent CVD, the composition of the pill aimed to decrease the incidence of four risk factors, namely BP, LDL-C, homocysteine, and platelet function [13]. Later on, based on their findings from a randomized, double-blind, placebo-controlled crossover trial of a Polypill among individuals aged 50 and older without a history of CVD, the authors proposed the application of the fixed-dose combination (FDC) protocol in individuals over 55 years, independent of their cardiovascular risk status, claiming that one-third of people taking the multi-drug pill after this age would benefit, gaining, on average, about 11 years of life free from an IHD event or stroke [7,95,96].

The scientific community strongly opposed this concept due to the uncertain ramifications of medicalizing an entire society, the expenses associated with probable bad reactions, the psychological impact on a healthy population, and the potential for fostering harmful lifestyle habits [97,98,99], suggesting a more targeted application of the Polypill as a main preventive measure in high-risk individuals without cardiovascular disease (CVD) [78]. Nevertheless, the positive outcomes of randomized clinical trials showing unequivocal evidence of benefits among diverse groups of individuals, as well as the good economic projections of the application of a fixed-dose strategy, provoked special interest. Many researchers view this approach as feasible in populations of developing countries, which, paradoxically, are those with the highest risk of developing fatal cardiovascular events [100]. Currently, there are several studies looking at the efficacy, security, and viability of the application of FDC therapy for the primary and secondary prevention of CVD in populations with medium and low income (MLI), where the age factor is under strict control by health professionals [101,102].

The issue of age and cardiovascular events has been discussed for decades due to the abundance of reports of ischemic heart episodes or stroke in younger subjects without CVD symptoms. The relevance of age to the development of CVD has already been addressed by the Prospective Studies Collaboration study [103], a meta-analysis of 61 cohorts recruited between 1950 and 1990. This study reported log-linear associations of systolic and diastolic BP with death from IHD and stroke in participants aged 40–89 years. Remarkably, the results showed that, whereas between middle and old age, BP related strongly and directly to vascular (and overall) mortality, there was no evidence of a risk reduction when BP values were under 115/75 mmHg. The authors of the study concluded that even if the starting level of risk is as low as 5% (calculated risk at 50 years old), the use of statins to prevent major CVD events in younger individuals whose CVD index levels are normal would be a worthy option, confirming the relevance of age for the therapeutic prevention of cardiovascular events [103].

Later, a longitudinal study based on the electronic health records from 1997 to 2010 from CALIBER (CArdiovascular research using LInked Bespoke studies and Electronic health Records) was performed [43]. To evaluate the age-specific heterogeneity of acute and chronic CVD and to estimate the lifetime risks (up to 95 years of age), they looked at the values of systolic and diastolic BP. The group assembled a cohort of 1.25 million patients 30 years of age or older and initially free from CVD. After 5.2 years of median follow-up, the group reported associations for 20/10 mm Hg changes in systolic/diastolic BP. Additionally, the researchers noted that the equivalence of these increases in cardiovascular disease (CVD) risk differed based on age (on average, a 20 mm Hg change in recorded initial CVD presentation of 83.098 cases) and gender (with men with hypertension having a higher incidence of myocardial infarction or fatal coronary heart disease and women with hypertension having a higher incidence of stroke). These findings have sparked significant controversy with respect to the advantages of treating mild (stage 1) hypertension in younger individuals in the absence of evidence of harm to specific organs and a relatively low 10-year risk of cardiovascular disease (CVD).

The evidence provided by new studies aimed at summarizing and discussing some of the most relevant clinical trials in epidemiology, diagnostics, and treatment of hypertension published in 2020 and 2021 [104] confirm previous data indicating a direct relationship between the age of onset of hypertension and risk for future CVD, in addition to stressing the importance of intensified BP control individually performed and analyzed according to the age of the patient. However, the authors claim that although age is a decisive factor in the level of risk of CVD, the management of hypertension, even in older patients, requires a comprehensive evaluation, even in high-risk patient groups, e.g., in the elderly (≥80 years). In this context, the protocol suggested by Wald et al. [7,105] to prevent the appearance of CVD in younger individuals without any known risk appears inadequate.

Additionally, given that adherence to treatment is a predicting factor for the success of a treatment, a qualitative study performed on patients’ views about taking a Polypill to manage cardiovascular risk based on age was executed [106]. The researchers documented patients who voiced apprehensions over the unnecessary prescription of Polypills for primary prevention, citing age as a distinct deciding factor and the potential for adverse consequences. They identified potential advantages, specifically for patients at high risk, but only with regular control of BP and cholesterol. These results coincide with other studies carried out in Spain and Belgium, which reported a high degree of satisfaction with the use of Polypills in patients but only under the supervision of a primary care physician based on a thorough evaluation of other factors in addition to age [107].

Since the effectiveness of CVD treatment depends on the compliance of the individual, the importance of his/her confidence in the protocols is fundamental. A study conducted by Hosein et al. [107] using only the criterion of age revealed that patients are skeptical about the benefits of a Polypill for the prevention of CVDs over risks. Hence, a prevention strategy using a Polypill after the age of 55 years needs to be supported by more studies in terms of benefits and risks associated with this approach, as well as patient education. Moreover, the investigation of newer and more efficient methods of early detection of asymptomatic CVD is fundamental to reach a wide consensus among physicians (and patients) on the use of the Polypill as an efficient method of prevention of the insurgence of CVDs.

Conversely, as a person ages, their risk of developing atherosclerosis increases. This risk is further elevated in individuals over 75 years of age who already have atherosclerotic occlusive disease, increasing their overall cardiovascular risk of ischemic events such as stroke, coronary events, limb ischemia, and renal failure. Additionally, elderly patients often have multiple comorbidities that require several medications. A single individual in this age range may need to take up to nine pills daily [108]. Polypharmacy has its disadvantages, including the lack of adherence, which is one of the most important leading causes of treatment failure. A physician may also be reluctant to prescribe multiple drugs because of the pharmacological interactions that may affect the drug’s action, its effect on the patient, the toxicity level, or both.

The effectiveness of administering a Polypill for primary prevention in vulnerable populations depends on various factors. These include the efficacy of statins, antihypertensives, and platelet aggregation inhibitors such as aspirin. Studies such as the ALLHAT-LLT, PROSPER, and JUPITER trials, as well as the Catalan database (SIDIAP), have demonstrated the crucial role of statins in diabetic individuals but not in those with low-to-moderate cardiovascular risk [44,62,109]. However, other studies involving a significant number of participants, such as a study involving 326,981 eligible veterans (mean age, 81.1 years) in the US without atherosclerotic cardiovascular disease, reported that the use of statins is associated with lower all-cause mortality [110].

Although novel Polypills offer the possibility to adjust their components, further studies are required to assess their effects on the elderly population, taking into account their specific clinical characteristics before administering a Polypill. Similarly, it is crucial to gather adequate information on the benefits of aspirin medication versus the risk of bleeding, as well as the optimal systolic and diastolic BP values in older patients with none-to-low cardiovascular risk, just like in the case of statins. Since newer Polypills contain one or two statins, low-dose aspirin, and one or two antihypertensive agents, the doctor needs to conduct a proper assessment of the elderly patient to determine the relevance of Polypill administration to avoid over-medication or undertreatment, especially in patients with frailty syndrome who are over 80 years of age.

Statins and low doses of aspirin can decrease symptoms of recurrent cerebral ischemia and improve kidney function in elderly individuals with comorbidities like diabetes or previous cardiovascular events. Statins are recommended for both symptomatic and asymptomatic PAD patients, whereas PCSK9 inhibitors are suggested for those who do not respond well to statins or have an ICAS of over 50% and dyslipidemia [111,112]. Aspirin is the primary antiplatelet agent recommended in patients with atherosclerosis when there is more than a 50% lumen reduction [113]. Regarding the BP of patients 80 years of age and older, target in-office pressure ranges of 130–139 mmHg and 70–79 mmHg for systolic and diastolic pressure, respectively, are recommended [22].

Despite the limited number of studies targeting the elderly, there is strong evidence of the positive effects of the administration of statins, aspirin, and antihypertensive agents to prevent fatal events in high-risk individuals. The Polypill has the advantage of providing all three medications while enhancing the outcomes observed with a single pill by improving the patient’s adherence to the pharmacological regime. As a consequence, older patients with antecedents of ischemic events are good candidates to take a Polypill, as it will only benefit them.

## 7. The Cardiovascular Polypill: Bioavailability of the Ingredients and Drug-Drug Interactions

A Polypill is a combination of drugs with fixed doses that effectively prevent cardiovascular diseases (CVDs). Clinical studies have identified two primary categories of Polypills: the single-purpose Polypill, which integrates various low-dose drugs to address a single CVD risk factor, such as high blood pressure or elevated serum cholesterol levels, and the multipurpose or cardiovascular Polypill. This type of Polypill includes three to four medicinal components, each with the capacity to mitigate a significant cardiovascular risk factor [18]. While the single-purpose pill for the primary and secondary prevention of hypertension is already approved by the FDA (Food and Drug Administration) and is on the market under certain brands, the multipurpose Polypill is not currently available for sale in the United States. However, one type of cardiovascular Polypill, the CNIC-Polypill composed of ramipril, atorvastatin, and low-dose aspirin is the only currently marketed cardiovascular Polypill in Europe.

In the last decade, researchers have developed different compositions of the cardiovascular Polypill for the prevention of CVD to test in clinical FDC therapy. The classic design of the pill includes drugs belonging to three main types, namely antihypertensives, statins, and inhibitors of platelet aggregation. Given the potential of this type of intervention in the prevention of cardiovascular events, multiple studies have been conducted on the dynamics of the components of the Polypill regarding their interactions that determine its efficacy and the possible adverse effects that such interplay can produce.

The pharmaceutical development of a cardiovascular Polypill presents unique challenges. For example, the selection of the type, number, and dose of active drugs and how they will be delivered depends on clinical, pharmaceutical, and commercial matters and how they are prioritized. There must be a careful and exhaustive gathering of information to present to the regulatory agencies before a particular Polypill can be approved for use in the general population [114,115]. While combining multiple active ingredients in a single dosage form would be a significant advancement in preventing cardiovascular conditions, it is crucial to thoroughly assess all the mentioned factors and create a well-planned development strategy to increase the likelihood of success.

Based on the findings of The Indian Polycap Study (TIPS) [101], a phase 2, double-blind, randomized trial performed to evaluate the effect of the Polycap on BP, lipids, heart rate, platelet aggregation, and tolerability, Patel et al. [116] performed a five-arm, randomized, single-dose, two-period, two-treatment, two-sequence crossover trial with at least a 2-week washout period in a total of 195 healthy volunteers to assess the bioavailability and drug–drug interactions of each ingredient of the mixed Polypill (Polycap™) used in the TIPS study. The Polycap™ consisted of aspirin, ramipril, simvastatin, atenolol, and hydrochlorothiazide, and it was selected because of its safety and effectiveness in reducing multiple cardiovascular risk factors in the participants.

The study did not find any evidence of pharmacokinetic drug–drug interactions between the components of the Polycap^TM^. Furthermore, the bioavailability of each ingredient of the pill was preserved. Moreover, the participants did not show serious adverse events, and the pill was well tolerated [116]. Interestingly, these results were reproduced by other randomized clinical trials that tested new multipurpose Polypills with varying constituents and the interactions with other drugs for the treatment of CVD but that are not included in the formulation of the Polypill. Such is the case of the study performed by Yusuf et al. [117] to evaluate the bioavailability and tolerability of two doses of the low-strength Polycap™ and to determine the tolerance and safety of incremental doses of potassium supplementation in individuals with multiple risk factors or stable CVD. Interestingly, the study showed a better reduction in CVD risk factors with two doses of low-strength Polycap™ (equivalent to one full dose) and good tolerance/safety of this Polypill with potassium supplementation [117].

The Polypill is still under evaluation and, like any drug, has certain limitations. As with the multi-drug treatment, the Polypill may not be suitable for everyone. Thus, β-blockers are contraindicated in individuals with asthma, ARBs, and ACE, and dual blockade can induce the development of acute renal failure and hyperkalemia [118], while statins, although in low percentages, can cause rhabdomyolysis [119]. Likewise, some people are intolerant to aspirin [120], while others are at risk of bleeding. However, the TIPS-3 randomized, controlled trial did show that in participants without CVD but with intermediate CV risk, combined treatment with a Polypill plus aspirin led to a lower incidence of cardiovascular events than the controls lacking aspirin in the composition, indicating the importance of the correct evaluation of risk/benefit for individuals before administration of a Polypill [113].

Although the studies performed thus far show that complications are not common and have rarely induced participants to abandon the trials, there are no studies to ascertain whether monitoring will avoid probable complications. More studies in this area are necessary before the safety of the Polypill and the diversity of formulations recently introduced will be systematically introduced for primary or secondary prevention purposes.

## 8. Efficacy and Tolerability of the Cardiovascular Polypill

Reports on the positive effects of the multipurpose Polypill as a novel approach for reducing CVD events by improving BP and hyperlipidemia have triggered wide interest in the potential of the combination of cardiovascular medications containing aspirin and agents to lower BP and cholesterol in a single pill. These clinical trials compared multipurpose Polypills with placebo or usual care to test the impact of FDC treatment on clinical outcomes, measured as clinical efficiency and adverse events of the specific Polypill, most of them with positive results.

In this context, the Pill Collaborative Group [121] conducted a randomized, double-blind, placebo-controlled Polypill trial in seven countries, namely Australia (n = 21), Brazil (n = 8), India (n = 109), the Netherlands (n = 102), New Zealand (n = 12), the United Kingdom (n = 113), and the United States (n = 13). The Polypill consisted of 75 mg aspirin, 10 mg lisinopril, 12.5 mg hydrochlorothiazide, and 20 mg simvastatin. The investigators administered the Polypill to 378 individuals for 12 weeks, without an indication for any component of it but with an estimated 5-year cardiovascular disease risk of over 7.5%. In this trial, Polypill treatment reduced systolic BP by 9.9 (95% CI: 7.7 to 12.1) mmHg and LDL-C by 0.8 (95% CI 0.6 to 0.9) mmol/L. The discontinuation rates due to adverse effects or other causes did not show statistical significance between the Polypill group (23%) and the placebo group (18%; *p* = 0.2) [121].

To further explore the effectiveness and tolerability of the multipurpose Polypill for both primary and secondary prevention of CVD, a new clinical trial analyzed the data of the Cochrane Central Register of Controlled Trials (CENTRAL) [122]. The criteria of selection included RCTs of fixed-dose combination therapy (Polypill), including at least one BP-lowering and one lipid-lowering component versus usual care, placebo, or a single-drug active component for any treatment duration in adults, without restrictions on the presence or absence of pre-existing CVD [122,123]. The risk was evaluated using the Cochrane risk of bias assessment tool [123].

The observations of nine RCTs (seven for primary prevention) with 7047 participants revealed mild adverse events in both the intervention (30%) and control (24%) groups, with participants randomized to FDC therapy being 20% more likely to report an adverse event. Nevertheless, none of those events cause the withdrawal of the patient from the trial, indicating a good tolerance to the pill [122].

The effectiveness and safety of the Polypill were also assessed in the PolyIran study [124], which was stratified in three districts of the Golestan province in Iran. The research was a two-group, pragmatic, cluster-randomized trial nested within the Golestan Cohort Study (GCS), a cohort study with 6838 individuals of both sexes aged 40–75 years. The composition of the Polypill included hydrochlorothiazide, aspirin, atorvastatin, and either enalapril or valsartan for primary and secondary prevention of CVD. Remarkably, both the number of participants and the design of the trial provided a better power to test the impact of a multipurpose, fixed-dose combination Polypill on clinical outcomes than other assessments [18].

In the clinical trial, the participants were divided into two groups based on their medical history. During the follow-up period, 8.8% of the patients receiving minimal care experienced major cardiovascular events, while the percentage was slightly lower for the Polypill group, at 5.9%. However, this difference was not statistically significant, regardless of whether the patients had pre-existing CVD. Both groups had similar rates of adverse events such as intracranial hemorrhages (10 participants in the Polypill group and 11 participants in the minimal care group) and upper gastrointestinal bleeding (13 participants in the Polypill group and 9 participants in the minimal care group) during the five-year follow-up. Nevertheless, there was no significant difference in safety between the two groups, as reported by 109 participants [124].

In 2022, González-Juanatey et al. conducted the NEPTUNO trial, a retrospective observational study aimed at evaluating a Polypill containing aspirin, ramipril, and atorvastatin in its formulation, the CNIC-Polypill [58]. This study proved, for the first time, that the use of a Polypill significantly reduced the incidence of recurrent MACE (secondary prevention) and delays the time to event in real-world patients with a history of ASCVD, as compared to three different active treatments, without increasing fatal events in the participants. The study was carried out on a large sample size, and its design, execution, and statistical analysis strengthen the case for the convenience of the Polypill strategy not only for primary but also for secondary cardiovascular prevention in clinical practice.

The SECURE trial, a phase 3 RCT multicentric study performed in 2499 elderly patients with MI in the previous 6 months, assessed the effectiveness of the CNIC-Polypill with respect to CV events and mortality in secondary cardiovascular prevention [79] at different doses according to the clinical and analytical parameters of each of the participants in the trial. The Polypill contained aspirin (100 mg), atorvastatin (20 or 40 mg), and ramipril (2.5, 5, or 10 mg). According to the data, the CNIC-Polypill group had a 33% lower rate of death from cardiovascular disease. This reduction was seen in all predefined subgroups, including those based on age, sex, diabetes, CKD, and prior cardiovascular events. At 24 months, the CNIC-Polypill group had a patient-reported drug adherence rate that was 17% higher than the standard care group, and both groups experienced similar side effects.

The SECURE trial demonstrated the safe application of a Polypill-based method, which significantly lowered cardiovascular events and death. Furthermore, the pharmaceutical treatment satisfaction questionnaire showed that after six months, the mean (±SD) global satisfaction score for 847 patients in the Polypill group and 818 patients in the usual-care group was 71.5 ± 18.1 and 67.7 ± 18.5, respectively. Global satisfaction scores at 24 months were 74.4 ± 17.5 and 67.8 ± 17.9, respectively. Irrespective of the trial participant’s affiliation, the researchers reported no adverse effects in the participants, and no patient withdrew from the study because of treatment-related side effects.

This new evidence suggests that adjusting the dose of ramipril or atorvastatin through the use of Polypill could prevent over- or under-medication and medical inertia. A fixed-dose Polypill taken once daily could be an essential part of an effective secondary prevention strategy. It simplifies treatment and improves accessibility and adherence to therapy for primary and secondary prevention of CVD.

## 9. The Polypill in the Management of Cardiovascular Disease in Medium- and Low-Income Countries and the Adherence to Long-Term Therapies

Globally, non-communicable diseases (NCDs) account for more than 60% of disability-adjusted life years (DALYs), 70% of deaths, and more than 80% of years lived with disability (YLD) [125]. CVD represents 24% of NCD-related DALYs, with ischemic heart disease and cerebrovascular disease as the two major causes of disability globally [126]. Despite a 14.5% decline in age-standardized CVD mortality rates globally between 2006 and 2016 [127], more than 80% of CVD deaths occur in LMICs, with a total economic loss of USD 3.7 trillion between 2011 and 2015, which represents half of the economic burden of non-communicable diseases (NCDs) and 2% of Gross Domestic Income (GDI) across LMICs. In these countries, CVD affects working-age populations much more than in high-income countries (HICs), with half of all cardiovascular deaths occurring in the 30–69 age group [126,127].

While CVD imposes a significant financial strain on healthcare systems, it is also among the most avoidable health issues. Implementing primary and secondary prevention strategies for cardiovascular disease (CVD) can effectively decrease the occurrence of cardiovascular events in both high-risk individuals and cardiovascular patients by addressing and managing modifiable risk factors such as blood pressure (BP) and cholesterol levels. Over time, numerous studies have shown that pharmacological therapy, which combines aspirin, statins, and antihypertensive medicines, is a highly effective form of prevention for high-risk persons (primary prevention) and cardiovascular patients (secondary prevention). Nevertheless, the lack of effective implementation of primary and secondary prevention can be attributed to non-adherence to treatment, which arises from factors such as the unavailability, under-prescription, unaffordability, and diversity of medications [128].

Adherence refers to how well patients stick to their prescribed medication regimen, as agreed upon with their healthcare practitioner (HCP) [129]. The lack of optimal implementation of primary and secondary prevention is primarily caused by the diversity, unavailability, under-prescription, therapeutic inertia, and affordability of drugs. This leads to non-adherence to treatment, increasing the risk of morbidity and mortality, resulting in higher healthcare costs and hospital admissions [75,130].

In a randomized, placebo-controlled, double-blind crossover trial with a Polypill formulated with three BP-lowering agents at half standard doses (2.5 mg amlodipine, 25 mg losartan, and 12.5 mg hydrochlorothiazide) and a standard dose of 40 mg simvastatin (manufactured by Cipla, India) [105], the authors found that 98% of participants took more than 85% of their allocated pills, while 24 out of 84 participants reported one or more symptoms associated with the pill (*p* = 0.01 compared to placebo), mainly referring to muscular ache, but none considered the symptoms troublesome enough to stop the treatment [105], indicating the relevance of such an approach and the importance of testing different compositions of the pill to determine the best combinations.

The World Health Organization (WHO) created the Multidimensional Adherence Model in 2003. This model aims to categorize the various reasons contributing to non-adherence to medicine. The model focuses on five dimensions, namely social/economic, patient, condition, therapy, and healthcare team- and system-related aspects [131]. Social and economic variables include inadequate health literacy, insufficient educational attainment, unemployment, and restricted social support. Factors associated with patients encompass their resources, knowledge, attitudes, and beliefs. Condition- and therapy-related aspects encompass elements related to the patient’s burden resulting from the illness itself and the administration of medications. These criteria are correlated with the primary challenges encountered by developing nations.

One interesting research study was performed by Soliman et al. [132] with the aim of evaluating the feasibility of the application of the Polypill protocol to standard clinical practice in low-income countries. The research was an open-label, parallel-group, randomized clinical trial involving three locations in Sri Lanka and enrolled a total of 216 patients without established CVD.

The authors compared the adherence to once-a-day Polypill (75 mg aspirin, 20 mg simvastatin, 10 mg Lisinopril, and 12.5 mg hydrochlorothiazide) with the adherence of individuals treated with the official protocol for CVD consisting of multiple pills per day. The study took place for three months. It was reported that 94% of the patients assigned to the Polypill group in low-income countries completed the program and returned for their three-month follow-up visits, suggesting that in developing countries, there is a high rate of patient acceptability with respect to monotherapy [132]. These results were confirmed by other clinical trials of FDC in participants from LMICs.

The findings of the UMPIRE trial indicated that on any day, patients who were still engaged with the drug-dosing regimen omitted about 10% of the scheduled doses; 42% of these omissions were of a single day’s dose, whereas 43% were part of a sequence of several days (“drug holidays”) [133]. Furthermore, the results indicated that one of the most important reasons for the failure of the traditional management of CVD was poor adherence of the patients to multi-drug therapies, leading to an escalation of cardiovascular events and a substantial increase in the costs of the overall health system.

To analyze the cost-effectiveness of administering a daily Polypill to prevent CVD (three antihypertensive drugs, a statin, and aspirin), the participants in the trial were selected among people with a risk of CVD equal to or greater than 15% over ten years in Latin America countries [49]. The authors found that at the end of the period of treatment, the lifetime risk of CVD was reduced by 15% in women and by 21% in men.

In this study, the researchers performed an estimate of the cost that the treatment with the Polypill could represent. The analysis showed that offering the Polypill to women at high risk and men aged fifty-five or older was the best approach to prevent major CV episodes in South American populations, with the potential to be cost-effective, especially in the Latin-American countries with the lowest gross national income [49]. This was the first time that gender was the variable of interest in Polypill trials, opening new perspectives for future studies, where gender will be specifically analyzed.

In a cost analysis study, the effectiveness of Polypill-based therapy was compared to standard care with separate drugs. The study used data from the Kanyini Guidelines Adherence with the Polypill GAP trial and linked Australian health service and medication administrative claims data. A comparison was made between the Polypill-treated group and those receiving usual care for people with established cardiovascular disease (CVD) or at a similarly high cardiovascular risk. The results revealed that the Polypill treatment group had a significantly lower mean annual pharmaceutical expenditure per patient when compared to usual treatment (*p* < 0.001; adjusted, eliminating Polypill cost) [134,135,136].

A Markov model cost–utility analysis was conducted to evaluate the cost-effectiveness of the CNIC-Polypill from the standpoint of the National Health System in Portugal [137]. Experts studied the NEPTUNO research, government reports, mortality tables, Portuguese registries, and literary works to determine efficacy, epidemiological costs, and utility data. The results included expenses per life year (LY) (calculated in 2020 euros) and acquired quality-adjusted LYs (QALYs). With the CNIC-Polypill, the model represents increases in the years patients would live and their quality of life. Compared to a combination of monocomponents, the researchers discovered that the CNIC-Polypill strategy for secondary prevention offers chances of 79.7% and 44.4 for the CNIC-Polypill to be cost-effective and cost-saving, respectively. Interestingly, the outcomes hold up well in sensitivity tests and are consistent in alternate scenarios. According to the researchers, the CNIC-Polypill more successfully reduces CV risk factors than monocomponents, making it a cost-effective preventive therapy for individuals with histories of heart disease or stroke [137].

These data suggest the convenience of an initiative-taking, guided management program to integrate the major number of medications associated with the prevention and treatment of CVD in both primary and secondary prevention settings. In this context, the application of FDC Polypill protocols to optimize the evolution and outcomes of patients with CVD appears to be an option to improve the adherence of patients to their medical regimens, increasing the efficacy of the treatment and significantly reducing costs.

These results open the possibility of introducing new guidelines based on FDC protocols for the primary and secondary treatment of patients with substantial risk of developing CVD or those already presenting with CVD, whose adherence to traditional medical treatments is poor, especially in populations of LMICs, independent of the country of residence [126]. Currently, there is enough evidence showing that the Polypill strategy is a desirable alternative in controlling CVD due to its high efficiency and safety, constituting a useful resource for prevention in populations at risk of CVD, especially in LMICs.

## 10. Discussion

CVD is a multifactorial disease that is a major cause of disability and premature death. For that reason, prevention and management have become a focus of healthcare providers (HCPs) worldwide. The latest Guidelines on the Primary Prevention of CVD emphasize the importance of the control of modifiable risk factors such as BP, blood cholesterol levels, smoking, diabetes, overweight or obesity, lack of physical activity, unhealthy diet, and stress through lifestyle adjustments [138]. However, this approach usually does not achieve satisfactory results, and major challenges remain.

Additionally, the tendency of CVD shows a notable decrease in developed countries, which is attributed to improvement in lifestyle-related risk factors. Since the year 2000, low- and middle-income countries (LMICs) have presented an increase in the number of CVDs [2]. The findings from two recent case–control studies, the INTERHEART [139] and INTERSTROKE [140] trials, showed that although high-income countries have the highest risk-factor burden, they present the lowest rates of major CVDs and death incidences compared with low-income countries. According to the authors, this might be explained by better control of risk factors and more frequent use of proven drug treatments and coronary interventions in high- and middle-income countries than in poorer countries [2].

The obvious conclusion of the above-mentioned studies is that many of the registered cardiovascular events could be avoided with the appropriate use of the drugs of proven efficiency recommended for secondary CVD prevention, namely aspirin, β-blockers, ACEi, ARBs, and statins [6]. However, analysis based on the data provided by the Prospective and Urban Rural Epidemiology (PURE) trial (2002–2030), an observational, prospective cohort study of 27 high-, middle-, and low-income countries, showed that the four drugs were potentially unaffordable for 0.14% of households in high-income countries, 25% of households in upper–middle-income countries, 33% of households in lower–middle-income countries, 60% of households in low-income countries (excluding India), and 59% of households in India [102]. Thus, differences in acquisition capacity are clear.

Cardiovascular Polypills containing aspirin, statins, and one or more antihypertensive medications, along with lifestyle interventions, represent an attractive, safe, and cost-effective strategy for primary and secondary prevention of CVD. The formulation and administration of the Polypill can overcome the difficulties faced by health professionals in the control of individuals with or without CDV with low to high risks, such as the lack of adherence to long-term therapies due to the multiple pills needed to be taken each day, the abandonment of treatment, and the lack of economic resources to face the cost of medicines.

Adherence to therapies is a primary determinant of treatment success. Poor adherence attenuates optimum clinical benefits and reduces the overall effectiveness of health systems. Poor adherence is related to the number of pills that the patient must take each day, the distinct types of medicines, and the time of the day at which each drug must be taken. It is well known that it is common for elderly, sick patients to take as many as 8 to 12 different pills per day. The Polypill is a single pill composed of the generic drugs commonly used for the treatment of CVD to be taken once a day, thus eliminating conflicts between the number of ingestions, the type of drug to be consumed, and the time of the day at which each medication needs to be taken. Therefore, the Polypill can simplify treatment and increase adherence.

Cost-effectiveness analysis of Polypills plays a key role in determining drug adherence. To date, several high-quality studies have assessed cost-effectiveness, most of them focusing on the economic burden of health care, with the cost of Polypills being one of the most principal factors. Systematic studies evaluating primary and secondary prevention of CVDs have proven that the use of a Polypill is a cost-saving technique compared to antihypertensive agents alone or antihypertensive agents plus statins. This is especially true for high-risk populations [11,134,141], such as those from medium- and low-income countries, as the magnitude and impact of poor adherence in developing countries are higher, given the inadequacy of health resources and inequities in access to health care. The increase in compliance due to Polypill treatment can lead to a decrease in morbidity and death due to CVD, with a better cost benefit [142].

An important obstacle with respect to the application of the Polypill is the feasibility of health professionals prescribing a Polypill for their patients, as well as the acceptance of the patients. Reports from semi-structured interviews performed to investigate personal perspectives towards prescribing and monitoring the outcomes of treatment with a Polypill [143] reveal that although most providers recognize that a Polypill would help improve their patients’ adherence to CVD medications because of the ease of use, convenience, and cost-savings of the Polypill, healthcare professionals are skeptical about the role of a Polypill.

General practitioners point out that the evidence presented based on multiple RCTs on unintended effects is insufficient or not reliable enough to support the hypothesis generated from the observational studies presented until now. They are particularly reluctant to prescribe a Polypill only based on age and feel that continuous monitoring of BP and cholesterol would be required. The inability to titrate the components of a Polypill for each patient is seen as a further disadvantage, although later FDC preparations are being considered in response to this issue. Finally, a considerable group of healthcare providers agreed that the Polypill formulations used in most trials are inappropriate for some secondary prevention patients because of the low and inflexible dosing. Rather, their position was that the Polypill protocol could be useful in high-risk primary prevention in clinically stable patients [144].

The reluctance of health providers regarding FDC treatments with the Polypill reflects their concerns about empirical evidence of the Polypill’s effectiveness and safety and uneasiness regarding medicalization. If a Polypill approach is introduced, healthcare professionals must be instructed about the potential benefits of a drug-based population approach to primary and secondary prevention [143] to ensure the patient a soft change from the traditional monocomponent treatment to the Polypill approach without risks for the patient, and to give the practitioner confidence to perform such a change.

This method can improve health outcomes more than the development of new medications. Nevertheless, even if the effectiveness of the Polypill strategy is equivalent to that of current care, the cost is likely to be reduced, and so is long-term adherence to medications [134,145], which further increases the potential of the all-in-one medication approach. Nevertheless, the issue of health practitioners’ willingness to adapt and embrace change and their patients’ acceptance remains a significant challenge change and acceptance of health practitioners and, hence, their patients.

The CNIC-Polypill is currently the only Polypill with regulatory approval in 28 countries for both primary and secondary prevention of cardiovascular disease (CVD) [79,99]. This Polypill is formulated under the same principles and components as the patented original Polypills, characterized by keeping the constituents of the pill from interacting chemically or physically while preserving all of their pharmacokinetic and biopharmaceutical qualities [58,99]. Numerous empirical investigations conducted globally have consistently demonstrated favorable tolerability, cost-effectiveness, and enhanced adherence, as evidenced by the intervention’s efficacy [136,146,147].

With such a body of evidence, it is important to offer a clear overview of the management options for patients with different levels of cardiovascular risk based on FDC therapies. When a clinician chooses to initiate or transition to a cardiovascular Polypill for patients with hypertension, dyslipidemia, advanced atherosclerotic disease, or a confirmed cardiovascular event, a typical challenge arises. The dosage of the current antihypertensive drugs and statins needs to be adjusted based on the patient’s hypertension grade and LDL-C level. These factors can vary significantly among patients with similar overall cardiovascular risk. This issue should not pose a challenge, as the healthcare provider can select from multiple types of the CNIC-Polypill that offer different dosages of the BP-lowering and statin constituents. This provides increased versatility in terms of prescribing and utilizing the medication.

The implementation of protocols and algorithms that consider many real-life clinical scenarios, understanding that people may vary in the severity of blood pressure and LDL-C levels that determine the cardiovascular risk of the patient. Furthermore, the targets for blood pressure (BP) and low-density lipoprotein cholesterol (LDL-C) differ between primary and secondary cardiovascular prevention. Additionally, the specific target may need to be more stringent based on the presence of cardiovascular risk factors and comorbidities [32,34]. In every scenario, adjusting the dosages of the Polypill constituents (ramipril and/or atorvastatin) is necessary. Additionally, in patients whose blood pressure (BP) or low-density lipoprotein cholesterol (LDL-C) levels do not meet the desired target, the inclusion of additional medications that lower blood pressure, such as calcium channel blockers (CCBs), diuretics, or lipid-lowering drugs, maybe necessary [70].

In light of the aforementioned factors, Coca et al. released a comprehensive guide for healthcare professionals outlining the transition from initial treatment to the CNIC Polypill for primary or secondary cardiovascular disease (CVD) prevention in individuals diagnosed with atherosclerotic disease. These patients are currently receiving multiple drugs or pills to address their cardiovascular risk factors and comorbidities [57]. However, further research is required to provide guidance and assurance to physicians when prescribing a Polypill.

An additional scenario that necessitates consideration is when the healthcare provider encounters patients who have previously encountered a clinical cardiovascular event as a result of atherosclerotic vascular disease and are susceptible to an additional cardiovascular event. The recommended approach for managing these patients entails the administration of a combination of aspirin; a statin; ACi (or ARB); and drugs specific to their comorbidities, such as β-blockers, mineralocorticoid receptor antagonists, diuretics, CCBs, glucose-lowering drugs, or other medications [70]. To switch to the Polypill, the healthcare provider must search for an equivalent effective daily dose of the current ACE inhibitor or ARB and statin.

The therapeutic interchangeability between RAS blockers, such as ACEIs or ARBs, as well as statins, enables this possibility. One possible course of action is to transition to the CNIC-Polypill in addition to specialized medications for comorbidities, as well as diuretics or CCB if necessary. Suppose the desired blood pressure (BP) and/or low-density lipoprotein cholesterol (LDL-C) targets do not occur despite the administration of maximal doses of atorvastatin and the current treatment for comorbidities. In that case, healthcare professionals may consider incorporating a low or standard dose of a third antihypertensive medication, such as ezetimibe or a proprotein convertase subtilisin-kexin type 9 inhibitor (PCSK9i), as advised by current guidelines [57].

The utilization of an FDC of generic versions of various drug classes for individuals with a significant risk of cardiovascular events in a single pill represents a potential approach that has the potential to streamline the medication regimen for both prescribers and patients. Additionally, this strategy can decrease expenses for healthcare providers and patients [11]. Subsequent research endeavors should prioritize the identification of patients who will derive the most significant advantage from the use of FDC medicines, the promotion of multiple Polypills with distinct constituents and dosages, and the formulation of innovative regulatory approaches to ensure widespread accessibility of these pills across all nations globally.

## 11. Conclusions and Future Directions

The cardiovascular Polypill is a concept that appeared for the first time in 2003, thanks to the studies performed by Wald and Law [7]. It is a drug combination consisting of a single drug product in pill form (i.e., tablet or capsule) that combines multiple active pharmaceutical ingredients as a strategy for primary and secondary prevention of CBDs. There are now data from many independent, large, and long-term trials in primary prevention showing that a combination of blood-pressure-lowering agents and statins at low doses reduces the risk of CVDs by about 38%, with the effects corresponding to a nearly 50% relative risk reduction when aspirin is included in the Polypill.

The latest RCT reported by Castellano and collaborators confirms that a Polypill consisting of statins, an ACEi, and ASA reduces CV events by 25% compared with usual care after MI in patients older than 55 years based on the increased adherence and safety of the pill and the derived therapeutic protocols [79]. Recently, Yusuf and collaborators calculated that using the Polypill with only a 50% population acceptance rate could be crucial in achieving the WHO’s Sustainable Development Goal of reducing deaths from non-communicable diseases by 30% globally by 2030 [148]. Thus, the Polypill seems to fulfill the requirements of a desirable alternative for both prevention and treatment of CVDs at a lower cost than traditional treatments.

Despite the proven efficacy, safety, and economic convenience of the Polypill protocol, pharmaceutical companies are still reluctant to invest in its development and testing. It is expected that the reported evidence and the WHO and ESC recommendations regarding the cardiovascular Polypill can overcome such resistance.

The Polypill, in combination with lifestyle changes, can produce even more benefits and be locally or generically manufactured to improve the cost/benefit ratio. Guided by pre-tested algorithms like the one proposed by Coca and collaborators, physicians can focus on managing individuals with more complex conditions and obtain better outcomes individually and globally.

Overall, the Polypill, combined with advice to improve lifestyles, can facilitate the implementation of worldwide programs to limit the consequences of CVDs, which are the new pandemic of our century.

## Figures and Tables

**Table 1:** Clinical trials of fixed-dose combinations in relation to blood pressure and cardiovascular risk.

SPONSOR/NUMBER CLINICAL TRIAL (NCT)	TRIAL NAME	STUDY DESIGN	INTERVENTIONS	PRIMARY OUTCOME/REFERENCE
Sponsor: Cancer Research UK (CRUK), Chief Scientist Office, Scottish Government Health Directorates (CSO), Engineering and Physical Sciences Research Council (EPSRC), Economic and Social Research Council (ESRC), National Institute for Health Research (NIHR), National Institute for Social Care and Health Research (NISCHR), and The Wellcome Trust.	Cardiovascular disease research using linked bespoke studies and electronic health records: CALIBER programme	Linkages of multiple electronic heath record sources	Linking Clinical Practice Research Datalink, Myocardial Ischaemia National Audit Project, Hospital Episode Statistics, and Office of National Statistics.	A standardized data model was utilized, transforming routine electronic health record data into research-ready and shareable formats. This involves defining and curating metadata for over 300 variables, encompassing categorical, continuous, and event data on risk factors, cardiovascular diseases (CVDs), and non-cardiovascular comorbidities [43].
**Sponsor:** AstraZeneca NCT00227318	TROPHY-Candesartan Cilexetil Long-term Hypertension Prevention Trial	Multicenter, randomized, double-blind study in untreated subjects aged 30 to 65 years with entry BPs of 130 to 139/≤89 or ≤139/85 to 89 mm Hg; the patients were followed up for 4 years.	Participants were randomly assigned to receive placebo or a fixed (16 mg once daily) dose of candesartan cilexetil (candesartan).	The TROPHY study found higher-than-expected hypertension rates but a sufficient sample size for evaluating primary hypertension prevention. Baseline characteristics of subjects were outlined, and innovative statistical methods were discussed. DOI: 10.1161/01.HYP.0000130174.70055.ca [30].
***Sponsor*** *: Grant from Sanofi Aventis Pharma GmbH, Germany*	The PHARAO study: prevention of hypertension with the angiotensin-converting enzyme inhibitor ramipril in patients with high-normal blood pressure	Prospective, randomized, controlled, multicenter trial according to the Prospective, Randomized, Open, Blinded Endpoint (PROBE) design. A total of 1008 participants with high–normal office blood pressure were randomized to a ramipril treatment group (n = 505) and a control group (n = 503). The patients were followed-up for 3 years.	The participants received ramipril at a dose of 1.5 mg once daily in the morning for 3 days, then 2.5 mg for 7 days and 5 mg thereafter.	A total of 155 patients (30.7%) in the ramipril group and 216 patients (42.9%) in the control group reached the primary endpoint, with a 34.4% relative risk reduction (*p* = 0.0001) in the ramipril group. Ramipril was more effective in reducing the incidence of manifest office hypertension in patients with high–normal blood pressure, but there was no significant difference in cerebrovascular and cardiovascular events between the two groups. Cough was more prevalent in the ramipril group (4.8% vs. 0.4%). doi:10.1097/HJH.0b013e3282ff8864 [31].
***Sponsor*****:** National Health and Medical Research Council, Australia ACTRN12616001144404	Ultra-low-dose quadruple combination blood pressure–lowering therapy in patients with hypertension: The QUARTET randomized controlled trial protocol	Multicenter, double-blind, parallel-group, randomized, phase 3 trial among Australian adults (≥18 years) with hypertension who were untreated or receiving monotherapy.	Participants were randomly assigned to either treatment, which started with the Quadpill (containing irbesartan at 37·5 mg, amlodipine at 1·25 mg, indapamide at 0·625 mg, and bisoprolol at 2·5 mg) or an indistinguishable monotherapy control (irbesartan 150 mg).	The intervention group had lower systolic blood pressure by 6.9 mm Hg and higher blood pressure control rates, at 76%, compared to the control group (58%). Adverse event-related treatment withdrawals at 12 weeks showed no significant difference between the groups. DOI: 10.1016/S0140-6736(21)01922-X [36].
***Sponsor*****:** National Heart, Lung, and Blood Institute (NHLBI) NCT00302718	The Antihypertensive and Lipid-Lowering Treatment to Prevent Heart Attack Trial (ALLHAT)	Randomized, double-blind, active-controlled clinical trial conducted from February 1994 through March 2002. A total of 33,357 participants aged 55 years or older with hypertension and at least one other CHD risk factor from 623 North American centers were included. Antihypertensive and lipid-lowering treatments were administered to prevent heart disease.	Participants were randomly assigned to receive chlorthalidone (12.5 to 25 mg/d, n = 15,255), amlodipine (2.5 to 10 mg/d, n = 9048), or lisinopril (10 to 40 mg/d, n = 9054) for planned follow-up of approximately 4 to 8 years. The subanalysis of the lipid lowering components of the ALLHAT (ALLHAT-LLT) was performed with the same population of the initial ALLHAT trial.	After five years, systolic blood pressures were significantly higher in the amlodipine group (0.8 mm Hg, *p* = 0.03) and the lisinopril group (2 mm Hg, *p* < 0.001) compared with chlorthalidone group, while diastolic blood pressure was significantly lower in association with amlodipine (0.8 mm Hg, *p* < 0.001). DOI: 10.1001/jama.288.23.2981 [41]. However, no benefit of primary prevention for all-cause mortality or cardiac events when pravastatin was initiated for adults 65 years and older with moderate hyperlipidemia and hypertension. doi:10.1001/jamainternmed.2017.1442 [44].
***Sponsor*****:** Grants from Servier, the Health Research Council of New Zealand, and the National Health and Medical Research Council of Australia	The perindopril protection against recurrent stroke study (PROGRESS)	Participants were randomly assigned to active treatment (n = 3051) or placebo (n = 3054) in a randomized, double-blind, placebo-controlled trial. A total of 6105 individuals from 172 centers in Asia, Australasia, and Europe were included.	Participants were randomly assigned to the following groups: active treatment (angiotensin-converting enzyme inhibitor perindopril [4 mg/d] for all patients, with the diuretic indapamide added at the discretion of treating physicians) or matching placebo(s).	Active treatment decreased major vascular events risk by 26%. Hypertensive and non-hypertensive subgroups showed similar reductions in stroke risk. Combination therapy (perindopril + indapamide) lowered blood pressure by 12/5 mm Hg and reduced stroke risk by 43%. Single-drug therapy decreased blood pressure by 5/3 mm Hg but did not reduce stroke risk. DOI:10.1016/S0140-6736(01)06178-5 [45].
***Sponsor*****:** National Health and Medical Research Council (NHMRC) of Australia and Servier International NCT00145925	Effects of a fixed combination of perindopril and indapamide on macrovascular and microvascular outcomes in patients with type 2 diabetes mellitus (the ADVANCE trial)	Randomized trial including over 10,000 patients with established type 2 diabetes in a factorial design. A total of 215 collaborating centers in 20 countries from Asia, Australia, Europe, and North America participated.	A total of 11,140 patients with type 2 diabetes mellitus were randomly assigned to a fixed combination of perindopril-indapamide (4/1.25 mg) or placebo.	Active treatment reduced the risk of death by 28% among patients with CCB at baseline compared to 5% among those without CCB. The risk reduction for death was 14% for the entire population. The relative risk reduction for major cardiovascular events was 12% for those with CCB at baseline and 6% for those without CCB at baseline. DOI: 10.1161/HYPERTENSIONAHA.113.02252 [46].
***Sponsor*****:** Grants from the British Heart Foundation and the Institut de Recherches Internationales Servier, and Imperial College London NCT00122811	The Hypertension in the Very Elderly Trial (HYVET)	Randomized, double-blind, placebo-controlled trial with the participation of 195 centers in 13 countries in Western and Eastern Europe, China, Australasia, and North Africa.	Participants received either the diuretic indapamide (sustained release, 1.5 mg) or a matching placebo. The angiotensin-converting enzyme inhibitor perindopril (2 or 4 mg) or a matching placebo was added if necessary to achieve the target blood pressure of 150/80 mm Hg.	Active treatment was linked with a 30% reduction in the rate of fatal or nonfatal stroke (95% confidence interval [CI], −1 to 51; *p* = 0.06), a 39% reduction in the rate of death from stroke (95% CI, 1 to 62; *p* = 0.05), a 21% reduction in the rate of death from any cause (95% CI, 4 to 35; *p* = 0.02), a 23% reduction in the rate of death from cardiovascular causes (95% CI, −1 to 40; *p* = 0.06), and a 64% reduction in the rate of heart failure (95% CI, 42 to 78; *p* < 0.001). DOI: 10.1056/NEJMoa0801369 [47].
***Sponsor*****:** AstraZeneca and CoordinatingCenteratSahl-grenskaUniversityHospital/Östra, Göteborg, Sweden Funded by the Canadian Institutes of Health Research and AstraZeneca; ClinicalTrials.gov number, NCT00468923	The Study on Cognition and Prognosis in the Elderly (SCOPE)	Multicenter, prospective, randomized, double-blind, parallel-group study designed to compare the effects of candesartan cilexetil and placebo in elderly patients with mild hypertension. A total of 4937 patients were recruited at 527 centers in 15 countries.	Participants received candesartan cilexetil 8 mgo.d. or a corresponding placebo o.d.; patients whose SBP remained > 160 mmHg or decreased by <10 mmHg since the randomization visit or whose DBP was >85 mmHg had their treatment doubled to two tablets (candesartan cilexetil, 16 mg o.d., or two matching placebo tablets o.d.)	Candesartan-based treatment decreased nonfatal stroke by 27.8% (95% CI, 1.3 to 47.2, *p* = 0.04) and all stroke by 23.6% (95% CI, −0.7 to 42.1, *p* = 0.056). No significant differences were observed in myocardial infarction and cardiovascular mortality. DOI: 10.1080/080370599439715 [48].
**Sponsor:** Canadian Institutes of Health Research and AstraZeneca NCT00468923	Heart Outcomes Prevention Evaluation-3 (HOPE-3)	Randomized, double-blind, placebo-controlled trial. A total of 228 centers in 21 countries were involved, using a 2 × 2 factorial design.	Participants were randomly assigned to receive daily administration of either a fixed-dose combination of candesartan at a dose of 16 mg and hydrochlorothiazide at a dose of 12.5 mg or placebo; participants were also randomly assigned to receive either rosuvastatin at a dose of 10 mg or placebo.	Participants in the subgroup for the upper third of systolic blood pressure (>143.5 mm Hg) who were in the active-treatment group had significantly lower rates of the first and second coprimary outcomes than those in the placebo group. DOI: 10.1377/hlthaff.2011.0948 [49].
**Sponsor:** National Heart, Lung, and Blood Institute (NHLBI) NCT00000514	Systolic Hypertension in the Elderly Program (SHEP)	A randomized, double-blind, placebo-controlled trial with an average follow-up of 4.5 years. A total of 4736 men and women aged 60 years or older with isolated systolic hypertension participate from 16 clinical centers in the United States.	Patients were randomly assigned to receive treatment with 12.5 mg/d of chlorthalidone (step 1) (either 25 mg/d of atenolol or 0.05 mg/d of reserpine (step 2) could be added) (n = 2365) or placebo (n = 2371).	The study analyzed the type, timing, and occurrence of first strokes and stroke fatalities, as well as changes in stroke incidence in participants who achieved a systolic blood pressure reduction of at least 20 mm Hg from baseline to below 160 mm Hg compared to those who did not. DOI: 10.1001/jama.284.4.465 [50].
**Sponsor:** Astra/Hassle, ICI Pharma, Merck Sharpe & Dohme (Sweden), Sandoz, and the Swedish County Councils	STOP-HYPERTENSION	Randomized, double-blind, intervention study involving a total of 116 health centers (out of 846) throughout Sweden and hypertensive men and women aged 70–84 years.	Participants receive treatment that consisted of 50 mg atenolol, 25 mg hydrochlorothiazide plus 2–5 mg amiloride, 100 mg metoprolol, or 5 mg pindolol.	Compared to placebo, active treatment significantly reduced the number of primary endpoints (94 vs. 58; *p* = 0.0031), as well as stroke morbidity and mortality (53 vs. 29; *p* = 0.0081). DOI: 10.1016/0140-6736(91)92589-T [51].
**Sponsor:** A grant from the Japan Heart Foundation NCT00134160	OlmeSartan and Calcium Antagonists Randomized (OSCAR) Study)	Multicenter, prospective, randomized, open-label, blinded end-point study. A total of 1164 Japanese elderly hypertensive patients were recruited to compare the efficacy of angiotensin II receptor blocker (ARB) up titration to an ARB plus calcium channel blocker (CCB) combination.	Participants were randomized to receive 20 mg/day olmesartan medoxomil as ARB monotherapy in the ‘Step 1’ period. If blood pressure was not adequately controlled and treatment was well tolerated, then the dose was changed to 40 mg/day olmesartan medoxomil in the high-dose ARB monotherapy group, or 20 mg/day olmesartan medoxomil and a CCB in the combination therapy group in the ‘Step 2’ period. At least 500 patients were enrolled in each group, and the follow-up duration was 3 years.	In patients with cardiovascular disease at baseline, there were more primary events observed in the high-dose angiotensin II receptor blocker group (HR, 1.63, *p* = 0.03); however, fewer events were noted in the subgroup without cardiovascular disease (HR, 0.52, *p* = 0.14). DOI: 10.1016/j.amjmed.2011.12.010 [52].
**Sponsor:** Novartis Pharmaceuticals NCT00549757	Aliskiren Trial in Type 2 Diabetes Using Cardiovascular and Renal Disease Endpoints (Core and Extension Phases) (ALTITUDE)	Randomized, double-blind, placebo-controlled, parallel-group study to determine whether, in patients with type 2 diabetes at high risk for cardiovascular and renal events, aliskiren, on top of conventional treatment, reduces cardiovascular and renal morbidity and mortality.	Participants were randomly assigned to receive aliskiren at a target dose of 300 mg once daily, in addition to conventional treatment, to lower the risk of illness and death caused by cardiac, circulatory, or kidney issues.	Systolic and diastolic blood pressures were lower with aliskiren (with between-group differences of 1.3 and 0.6 mm Hg, respectively), and there was a greater mean reduction in the urinary albumin-to-creatinine ratio (with a between-group difference of 14 percentage points; 95% CI, 11 to 17). DOI: 10.1056/NEJMoa1208799 [53].
**Sponsor:** The NHLBI, Duke University NCT02816736	EntrestoTM (LCZ696) In Advanced Heart Failure (LIFE Study) (HFN-LIFE)	A double-blind, randomized clinical trial was conducted; a total of 335 patients with advanced heart failure were included.	Participants were randomized to receive sacubitril/valsartan (target dose, 200 mg twice daily) or valsartan (target dose, 160 mg twice daily) in addition to recommended therapy.	Sacubitril/valsartan treatment did not show significant improvement in the clinical composite compared to valsartan, except for a statistically significant increase in non-life-threatening hyperkalemia. No other safety concerns were noted. doi:10.1001/jamacardio.2021.4567 [54].
**Sponsor:** Pfizer	The Anglo-Scandinavian Cardiac Outcomes Trial (ASCOT)- a blood pressure lowering arm (BPLA)	Multicenter randomized trial with a 2 × 2 factorial design. A total of 8580 UK-based patients were included.	Participants were randomized, using a 2 × 2 factorial design, to the BP-lowering arm (BPLA), receiving either amlodipine (4305)- or atenolol (4275)-based treatment. Of these, 4605 (54%) patients with total cholesterol ≤65 mmol/L and with no previous lipid-lowering treatment were further randomized to either atorvastatin (2317) or placebo (2288) in the lipid-lowering arm (LLA). The remaining 3875 patients formed the non-LLA group.	Patients treated with amlodipine had significantly fewer deaths due to stroke compared to those treated with atenolol. Among non-LLA patients, amlodipine-based treatment was associated with fewer cardiovascular deaths. In the LLA group, patients treated with statin had significantly fewer cardiovascular deaths. DOI: 10.1016/S0140-6736(18)31776-8 [42].
**Sponsor:** Novartis NCT00170950	Avoiding Cardiovascular Events Through Combination Therapy in Patients Living With Systolic Hypertension (ACCOMPLISH)	Prospective, multinational, multicenter trial including a total of 11,506 patients with hypertension who were at high risk for cardiovascular events.	Patients were randomly assigned to receive a combination of either benazepril and amlodipine or benazepril and hydrochlorothiazide. Both groups started with a daily dose of 20 mg of benazepril, followed by an increase to 40 mg after one month. The dosage of amlodipine or hydrochlorothiazide was adjusted as needed to achieve a target blood pressure of less than 140/90 mm Hg or 130/80 mm Hg for patients with diabetes or kidney disease.	The benazepril–amlodipine group had 9.6% primary-outcome events compared to 11.8% in the benazepril-hydrochlorothiazide group. The benazepril–amlodipine therapy resulted in a 2.2% absolute risk reduction and a 19.6% relative risk reduction. DOI:10.1016/S0895-7061(03)00603-4 [55].

## Data Availability

Not applicable.

## Supplementary Materials

The following supporting information can be downloaded at: https://www.mdpi.com/article/10.3390/jcm13113179/s1, Supplementary Material S1: Checklist for narrative, expert opinion, and textual evidence.
