# Supplementary material for: The Polypill: A New Alternative in the Prevention and Treatment of Cardiovascular Disease
Source: J Clin Med. 2024 May 29;13(11):3179. doi: 10.3390/jcm13113179 (PMC11172978; doi:10.3390/jcm13113179)
Supplement: Supplementary file 1 [file jcm-13-03179-s001.zip › jcm-2931764-supplementary.pdf]

SUPPLEMENTARY MATERIAL 1 (S1): Checklist for narrative, expert opinion, and textual evidence

ARTICLES TITLE	INCLUYE
A cardiovascular polypill for secondary stroke prevention in a tertiary centre in Ghana (SMAART): a phase 2 randomised clinical trial	Yes
A Polypill Strategy to Improve Global Secondary Cardiovascular Prevention: From Concept to Reality	Yes
A protocol for an economic evaluation of a polypill in patients with established or at high risk of cardiovascular disease in a UK NHS setting: RUPEE (NHS) study.	Yes
A review of polypills for the prevention of atherosclerotic cardiovascular disease	Yes
A polypill strategy to improve global secondary cardiovascular prevention: from concept to reality.	Yes
Acceptance of a Polypill approach to prevent cardiovascular disease among a sample of U.S. physicians.	Yes
Adoption and scale-up of the cardiovascular Polypill: a realist institutional analysis.	Yes
Association of polypill therapy with cardiovascular outcomes, mortality, and adherence: A systematic review and meta-analysis of randomized controlled trials.	Yes
Barriers and Facilitators to the Use of Cardiovascular Fixed-Dose Combination Medication (Polypills) in Andhra Pradesh, India: A Mixed-Methods Study.	Yes
Benefits of the Polypill on Medication Adherence in the Primary and Secondary Prevention of Cardiovascular Disease: A Systematic Review.	Yes
Correction: Cost-effectiveness of fixed-dose combination pill (Polypill) in primary and secondary prevention of cardiovascular disease: A systematic literature review.	Yes
Correction: Effectiveness of Polypill for Prevention of Cardiovascular Disease (PolyPars): Protocol of a Randomized Controlled Trial.	Yes
Correction: The use of the CNIC-Polypill in real-life clinical practice: opportunities and challenges in patients at very high risk of atherosclerotic cardiovascular disease - expert panel meeting report.	Yes
Correlation of polypill and blood pressure level: A systematic review of clinical trials.	Yes
Cost-benefit analysis of the polypill in the primary prevention of myocardial infarction and stroke.	Yes
Cost-effectiveness analysis of use of a polypill versus usual care or best practice for primary prevention in people at high risk of cardiovascular disease.	Yes
Cost-effectiveness of a fixed dose combination (polypill) in secondary prevention of cardiovascular diseases in India: Within-trial cost-effectiveness analysis of the UMPIRE trial.	Yes
Cost-effectiveness of a fixed-dose combination pill for secondary prevention of cardiovascular disease in China, India, Mexico, Nigeria, and South Africa: a modelling study.	Yes
Cost-effectiveness of the CNIC-Polypill versus separate monocomponents in cardiovascular secondary prevention in Spain	Yes
Economic Burden Associated with the Treatment with a Cardiovascular Polypill in Secondary Prevention in Spain: Cost-Effectiveness Results of the NEPTUNO Study.	Yes
[illegible] (polypill) [illegible] with cardiovascular disease or at high risk: A prospective, individual patient data meta-analysis of 3140 patients in six countries.	Yes

Effectiveness of Polypill for Prevention of Cardiovascular Disease (PolyPars): Protocol of a Randomized Controlled Trial.	Yes
Effects of a Polypill, Aspirin, and the Combination of Both on Cognitive and Functional Outcomes: A Randomized Clinical Trial.	Yes
Exercise is the real polypill.	Yes
Frontiers of cardiovascular polypills: From atherosclerosis and beyond.	Yes
Modular Titratable Polypills for Personalized Medicine and Simplification of Complex Medication Regimens.	Yes
Multifactorial Prevention of Cardiovascular Disease in Patients with Hypertension: the Cardiovascular Polypill.	Yes
Polypill Therapy, Subclinical Atherosclerosis, and Cardiovascular Events—Implications for the Use of Preventive Pharmacotherapy: MESA (Multi-Ethnic Study of Atherosclerosis)	Yes
Polypill use for the prevention of cardiovascular disease: A position paper	Yes
Polypill Brings Benefits to Patients with Cardiovascular Disease, Both Improving Medication Adherence and Demonstrating the Concept of Chronotherapy [Letter].	Yes
Polypill eligibility and equivalent intake in a Swiss population-based study.	Yes
Polypill for atherosclerotic cardiovascular disease prevention in Haiti: Eligibility estimates in a low-income country.	Yes
Polypill for Population-Level Primary Cardiovascular Prevention in Underserved Populations-A Social Epidemiology Counterargument.	Yes
Polypill for the prevention of cardiovascular disease (PolyIran): study design and rationale for a pragmatic cluster randomized controlled trial.	Yes
Polypill for Cardiovascular Disease Prevention in an Underserved Population.	Yes
Polypill in cardiovascular disease prevention: recent advances.	Yes
Polypill Programs to Prevent Stroke and Cut Costs in Low Income Countries: Moving From Clinical Efficacy to Pragmatic Implementation.	Yes
Polypill Therapy for Cardiovascular Disease Prevention and Combination Medication Therapy for Hypertension Management.	Yes
Polypill therapy, subclinical atherosclerosis, and cardiovascular events-implications for the use of preventive pharmacotherapy: MESA (Multi-Ethnic Study of Atherosclerosis).	Yes
Polypill use for the prevention of cardiovascular disease: A position paper.	Yes
Polypill with or without Aspirin in Persons without Cardiovascular Disease.	Yes
Polypill: an affordable strategy for cardiovascular disease prevention in low-medium-income countries.	Yes
Polypills for Primary Prevention of Cardiovascular Disease: A Systematic Review and Meta-Analysis.	Yes
Polypills for the secondary prevention of cardiovascular disease: effective in improving adherence but are they safe?	Yes
Polypills: Essential Medicines for Cardiovascular Disease Secondary Prevention?	Yes

Polypill to fight cardiovascular disease: birthday present was much appreciated.	Yes
Polypill to fight cardiovascular disease: cost effectiveness of statins for primary prevention of cardiovascular events is questionable.	Yes
"Polypill" to fight cardiovascular disease: interpretation of trial data is optimistic.	Yes
"Polypill" to fight cardiovascular disease: old joke has element of truth.	Yes
"Polypill" to fight cardiovascular disease: universal polypharmacy goes against recent beliefs in prescribing practice.	Yes
Practical Applications for Single Pill Combinations in the Cardiovascular Continuum.	Yes
Practical Decision Algorithms for the Use of the Cardiovascular Polypill in Secondary Prevention in Europe.	Yes
Prevention of Hypertensive Disorders of Pregnancy: a Novel Application of the Polypill Concept.	Yes
Process evaluation of the impact and acceptability of a polypill for prevention of cardiovascular disease.	Yes
Projected impact of polypill use among US adults: Medication use, cardiovascular risk reduction, and side effects.	Yes
Reaching blood pressure guideline targets with the CNIC polypill in patients with a previous cardiovascular event in Mexico: a post hoc analysis of the SORS study.	Yes
Reaching cardiovascular prevention guideline targets with a polypill-based approach: a meta-analysis of randomised clinical trials.	Yes
Real-life effect on the control of risk factors associated with initiation of the cardiovascular polypill created from equipotent drugs	Yes
Recruiting equal numbers of indigenous and non-indigenous participants to a 'polypill' randomized trial.	Yes
Safety and efficacy of a cardiovascular polypill in people at high and very high risk without a previous cardiovascular event: the international VULCANO randomised clinical trial.	Yes
Starting the polypill: the use of a single age cut-off in males and females.	Yes
Statins alone or polypill for primary prevention of cardiovascular diseases.	Yes
Strengths and Limitations of Using the Polypill in Cardiovascular Prevention.	Yes
The cardiovascular polypill: clinical data and ongoing studies	Yes
The cardiovascular polypill as baseline treatment improves lipid profile and blood pressure regardless of body mass index in patients with cardiovascular disease. The Bacus study.	Yes
The CNIC-polypill improves atherogenic dyslipidemia markers in patients at high risk or with cardiovascular disease: Results from a real-world setting in Mexico	Yes
The Concept of the Polypill in the Prevention of Cardiovascular Disease	Yes
The Effect of a Cardiovascular Polypill Strategy on Pill Burden.	Yes
The Effectiveness of Polypill for the Prevention of Cardiovascular Disease: A Meta-Analysis of Randomized Controlled Trials.	Yes

The efficacy and tolerability of 'polypills': meta-analysis of randomised controlled trials.	Yes
The Fuster-CNIC-Ferrer Cardiovascular Polypill: a polypill for secondary cardiovascular prevention	Yes
The Polymeal: a more natural, safer, and probably tastier (than the Polypill) strategy to reduce cardiovascular disease by more than 75%.	Yes
The use of the CNIC-Polypill in real-life clinical practice: opportunities and challenges in patients at very high risk of atherosclerotic cardiovascular disease - expert panel meeting report.	Yes
The polypill and the prevention of heart attacks and strokes by Caroline Telfer.	Yes
The polypill and cardiovascular disease.	Yes
The polypill approach - An innovative strategy to improve cardiovascular health in Europe.	Yes
The polypill in the primary prevention of cardiovascular disease: cost-effectiveness in the Dutch population.	Yes
Use of cardiovascular polypills for the secondary prevention of cerebrovascular disease	Yes
A 'polypill' aimed at preventing cardiovascular disease could prove highly cost-effective for use in Latin America. *Health affairs (Project Hope)* **2013**, *32* , 155-164, doi:10.1377/hlthaff.2011.0948.	Yes
A practical approach to switch from a multiple pill therapeutic strategy to a polypill-based strategy for cardiovascular prevention in patients with hypertension. *J Hypertens* **2020**, *38* , 1890-1898, doi:10.1097/hjh.0000000000002464.	Yes
Comparison of risk factor reduction and tolerability of a full-dose polypill (with potassium) versus low-dose polypill (polycap) in individuals at high risk of cardiovascular diseases: the Second Indian Polycap Study (TIPS-2) investigators. *Circulation. Cardiovascular quality and outcomes* **2012**, *5* , 463-471, doi:10.1161/circoutcomes.111.963637.	Yes
Cost-effectiveness of the polypill versus risk assessment for prevention of cardiovascular disease. *Heart* **2017**, *103* , 483-491, doi:10.1136/heartjnl-2016-310529.	Yes
Effectiveness of polypill for primary and secondary prevention of cardiovascular diseases (PolyIran): a pragmatic, cluster-randomised trial. *Lancet* **2019**, *394* , 672-683, doi:10.1016/s0140-6736(19)31791-x.	Yes
Effects of a polypill (Polycap) on risk factors in middle-aged individuals without cardiovascular disease (TIPS): a phase II, double-blind, randomised trial. *Lancet* **2009**, *373* , 1341-1351, doi:10.1016/s0140-6736(09)60611-5.	Yes
Implementation of a simple age-based strategy in the prevention of cardiovascular disease: the Polypill approach. *Journal of evaluation in clinical practice* **2012**, *18* , 612-615, doi:10.1111/j.1365-2753.2011.01637.x.	Yes
Issues to consider in the pharmaceutical development of a cardiovascular polypill. *Nature Clinical Practice Cardiovascular Medicine* **2009**, *6* , 112-119.	Yes
Polypill for cardiovascular disease prevention in an underserved population. *New England Journal of Medicine* **2019**, *381* , 1114-1123.	Yes
Polypill in cardiovascular disease prevention: recent advances. *Pol Arch Intern Med* **2023**, *133* , doi:10.20452/pamw.16460.	Yes
Polypill Strategy in Secondary Cardiovascular Prevention. *New England Journal of Medicine* **2022**, *387* , 967-977, doi:10.1056/NEJMoa2208275.	Yes
Polypill with or without Aspirin in Persons without Cardiovascular Disease. *The New England journal of medicine* **2021**, *384* , 216-228, doi:10.1056/NEJMoa2028220.	Yes
Preservation of bioavailability of ingredients and lack of drug-drug interactions in a novel five-ingredient polypill (polycap): a five-arm phase I crossover trial in healthy volunteers. *Am J Cardiovasc Drugs* **2010**, *10* , 95-103, doi:10.2165/11532170-000000000-00000.	Yes
Putting polypills into practice: challenges and lessons learned. *The Lancet* **2017**, *389* , 1066-1074.	Yes
The cardiovascular polypill: clinical data and ongoing studies. International Journal of Cardiology 2015, 201, S8-S14, doi:http://dx.doi.org/10.1016/S0167-5273(15)31027-5.	Yes

The polypill: from concept and evidence to implementation. *The Lancet* **2022**, *400* , 1661-1663.	Yes
Usefulness of a cardiovascular polypill in the treatment of secondary prevention patients in Spain: a cost-effectiveness study. *Revista Española de Cardiología (English Edition)* **2017**, *70* , 42-49.	Yes
"Polypill" to fight cardiovascular disease: now who's playing God?	n/a
“Polypill” to fight cardiovascular disease: Now who's playing God? *BMJ (Clinical research ed.)* **2003**, *327* , 807.	n/a
"Polypill" to fight cardiovascular disease: patients before populations.	n/a
“Polypill” to fight cardiovascular disease: Patients before populations. *BMJ (Clinical research ed.)* **2003**, *327* , 807.	Yes
The feasibility of polypill for cardiovascular disease prevention in Asian Population. *The Journal of Clinical Hypertension* **2021**, *23* , 545-555.	Yes
The feasibility of polypill for cardiovascular disease prevention in Asian Population.	Yes
Would primary healthcare professionals prescribe a polypill to manage cardiovascular risk? A qualitative interview study. *BMJ Open* **2013**, *3* , doi:10.1136/bmjopen-2012-002498.	Yes
Would primary healthcare professionals prescribe a polypill to manage cardiovascular risk? A qualitative interview study.	Yes
A polypill strategy to improve adherence: results from the FOCUS project. *Journal of the American College of Cardiology* **2014**, *64* , 2071-2082.	Yes
o A Polypill Strategy to Improve Adherence: Results From the FOCUS Project	Yes
A Polypill for primary prevention of cardiovascular disease: a feasibility study of the World Health Organization. *Trials* **2011**, *12* , 3, doi:10.1186/1745-6215-12-3.	n/a
A Polypill for primary prevention of cardiovascular disease: a feasibility study of the World Health Organization.	n/a
An international randomised placebo-controlled trial of a four-component combination pill ("polypill") in people with raised cardiovascular risk.	Yes
An international randomised placebo-controlled trial of a four-component combination pill ("polypill") in people with raised cardiovascular risk. *PLoS One* **2011**, *6* , e19857, doi:10.1371/journal.pone.0019857.	Yes
Cost-effectiveness and public health benefit of secondary cardiovascular disease prevention from improved adherence using a polypill in the UK. *BMJ Open* **2015**, *5* , e007111, doi:10.1136/bmjopen-2014-007111.	Yes
Cost-effectiveness of fixed-dose combination pill (Polypill) in primary and secondary prevention of cardiovascular disease: A systematic literature review. *PloS one* **2022**, *17* , e0271908.	Yes
Cost-Effectiveness of the CNIC-Polypill Strategy Compared With Separate Monocomponents in Secondary Prevention of Cardiovascular and Cerebrovascular Disease in Portugal: The MERCURY Study. *J Health Econ Outcomes Res* **2022**, *9* , 134-146, doi:10.36469/001c.39768.	Yes
Patients’ views about taking a polypill to manage cardiovascular risk: a qualitative study in primary care. *The British Journal of General Practice* **2015**, *65* , e447-e453, doi:10.3399/bjgp15X685657.	Yes
Randomized Polypill crossover trial in people aged 50 and over. *PLoS One* **2012**, *7* , e41297, doi:10.1371/journal.pone.0041297.	Yes
The CNIC-Polypill reduces recurrent major cardiovascular events in real-life secondary prevention patients in Spain: The NEPTUNO study. *Int J Cardiol* **2022**, *361* , 116-123, doi:10.1016/j.ijcard.2022.05.015.	Yes
The polypill: at what price would it become cost effective? *Journal of Epidemiology and Community Health* **2006**, *60* , 213-217, doi:10.1136/jech.2005.040253.	Yes

**CHECKLIST KEY**	**RESPONSES**

1. Is the generator of the narrative a credible or appropriate source?	YES/No/n/a
2. Is the relationship between the text and its context explained?	YES/No/n/a
3. Does the narrative present the events using a logical sequence so the reader or listener can understand how it unf	YES/No/n/a
4. Do you, as reader or listener of the narrative, arrive at similar conclusions to those drawn by the narrator?	YES/No/n/a
5. Do the conclusions flow from the narrative account?	YES/No/n/a
6. Do you consider this account to be a narrative?	YES/No/n/a

Ref. : McArthur A, Klugarova J, Yan H, Florescu S. Innovations in the systematic review of text and opinion. Int J Evid Based Healthc. 2015;13(3):188–195.
